# Supplementary material for: The impact of methane leakage on the role of natural gas in the European energy transition
Source: Nat Commun. 2023 Sep 16;14:5756. doi: 10.1038/s41467-023-41527-9 (PMC10505150; doi:10.1038/s41467-023-41527-9)
Supplement: Supplementary file 1 — Supplementary Information [file 41467_2023_41527_MOESM1_ESM.pdf]

Supplementary Information

Behrang Shirizadeh [1,2 *], Manuel Villavicencio [1], Sebastien Douguet [1], Johannes Trüby [1], Charbel Bou Issa [1] , Gondia Sokhna Seck [3], Vincent D'herbemont [3], Emmanuel Hache [3], Louis-Marie Malbec [3], Jerome Sabathier [3], Malavika Venugopal [4], Fanny Lagrange [4], Stephanie Saunier [4], Julian Straus [5] and Gunhild A. Reigstad [5]

[1] Deloitte Economic Advisory, 6 Place de La Pyramide Tour Majunga Deloitte, 92800, Puteaux, France.
[2] CIRED, 45 bis avenue de La Belle Gabrielle, 94736 Nogent sur Marne Cedex, France.
[3] IFP Energies Nouvelles, 1-4 Avenue Bois Preau, 92852, Rueil-Malmaison, France.
[4] Carbon Limits, C. J. Hambros plass 2, 0164 Oslo, Norway.
[5] SINTEF Energy Research, Sem Sælands Vei 11, 7034, Trondheim, Norway.
*Corresponding author: bshirizadeh@deloitte.fr, 33 (0)1 58 37 05 90

Supplementary Information 1 – The MIRET-EU model

Principles and the methodology

MIRET-EU is a multiregional and inter-temporal partial equilibrium model of the European energy system developed by IFPEN, based on the TIMES model generator. A complete description of the TIMES model equations appears in the ETSAP (Energy Technology Systems Analysis Program) documentation. It is a bottom-up techno-economic model that estimates the energy dynamics by minimising the total discounted cost of the system over the selected multi-period time horizon through powerful linear programming optimisers. The components of the system cost are expressed on an annual basis while the constraints and investment variables are linked to a period. Special care is taken to precisely track cash flows related to process investments and dismantling for each year of the horizon. The total cost is an aggregation of the total net present value of the stream of annual costs for each of the countries modelled. It constitutes the objective function (Eq. 1) to be minimised by the model in its equilibrium computation. A detailed description of the objective function equation is provided in Part II of the TIMES documentation[1]. We limit our description to giving general indications on the annual cost elements contained in the objective function:

- Investment costs incurred for processes,
- Fixed and variable annual costs,
- Costs incurred for exogenous imports and revenues from exogenous exports,
- Delivery costs for required commodities consumed by processes,
- Taxes and subsidies associated with commodity flows and process activities or investments.

$$NPV = \sum_{r=1}^{R} \sum_{y \in YEARS} (1 + d_{r,y})^{REFYR-y} * ANNCOST(r, y) \qquad (1)$$

NPV is the net present value of the total cost for all regions (the objective function),

ANNCOST(r,y) is the total annual cost in region *r* and year *y* (more details in section 6.2 of PART II[1]

$\boldsymbol{d_{r,y}}$ is the general discount rate,

REFYR is the reference year for discounting,

YEARS is the set of years for which there are costs, including all years in the horizon, plus past years (before the initial period) if costs have been defined for past investments, plus a number of years after end of horizon (EOH) where some investment and dismantling costs are still being incurred, as well as Salvage Value,

R is the set of regions/countries in the area of study.

The detailed energy system model (MIRET-EU) represents the European energy system divided into 27 European countries, including 24 EU Member States and 3 Non-EU countries (Norway, Switzerland and the United Kingdom), as described in Supplementary Figure 1. Each country has its own energy system with its main demand sectors. Moreover, each country can trade petroleum products, electricity, natural gas, hydrogen and CO_2 captured. Thus, the model fully describes within each country all existing and future technologies, from supply (primary resources), through the different conversion steps, up to end-use demands. It is set up to explore the development of its energy system from 2010 through to 2050 with 10-year time-steps. The 2010-2020 period is calibrated using up-to-date constraints (COVID-19 pandemic, Russia-Ukraine war, etc.) and the latest data provided by energy statistics databases such as the JRC-IDEES, POTEnCIA, EUROSTAT, and other international databases from IEA, IRENA and World Bank, among others. For clarity in the final charts, we set the reference year to the previous mid-decade (i.e., 2016) for benchmarking comparisons with existing data in all sectors and all countries. The significance of the findings rests in the development of the European energy system in 2030 considering the current policies and latest political measures, but also beyond, in the year 2050.

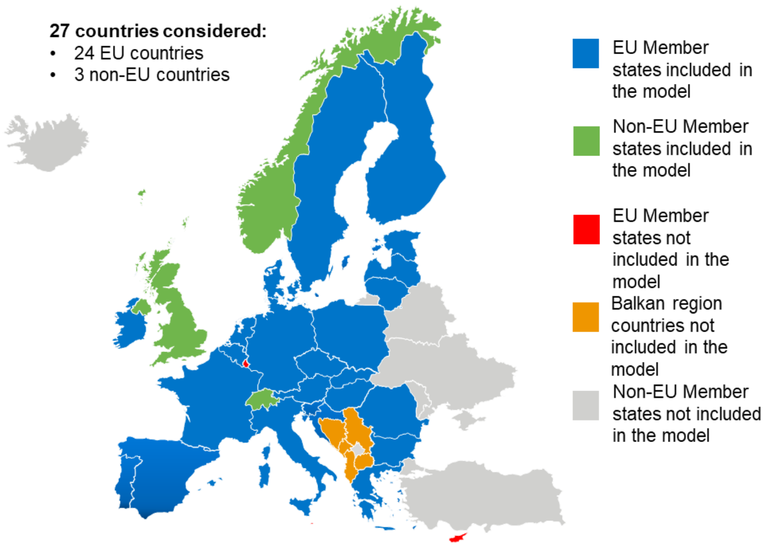

Supplementary Figure 1. Geographic coverage of the MIRET-EU model. The model includes EU 27, the UK and Norway, but it doesn't take Balkans and Cyprus into account[2].

MIRET-EU considers four seasons (spring, summer, autumn, winter) which are disaggregated into day, night, and peak resolution (following the same time slice disaggregation as in the world multiregional model TIAM-IFPEN). Every year is therefore divided in twelve time-slices that represent an average of day, night, and peak demand for each season of the year (e.g., summer day, summer night and summer peak, etc.).

The MIRET-EU model is data driven (parameter assumptions, technology characteristics, projections of energy service demands, etc.), its parameterisation refers to technology characteristics, resource data, projections of demand for energy services, policy measures, among other. This means that the model varies according to the data inputs while providing results such as technology pathways or changes in trade flows for policy recommendations. For each country, the model includes detailed descriptions of numerous technologies, logically interrelated in a Reference Energy System – the chain of processes that transform, transport, distribute and convert energy into services from primary resources and raw materials to the energy services needed by end-use sectors.

A few models have already been developed at European scale using the TIMES model over the last 15 years. The Pan-European TIMES (PET) model has been developed by the Kanlo team following a series of European Commission (EC) funded projects (NEEDS, RES2020, REACCESS, REALISEGRID, COMET, Irish-TIMES) between 2004 and 2010. It represents the energy system of 36 European regions. The JRC-EU-TIMES model is one of the models currently pursued and developed in the Joint Research Centre (JRC) of the European Commission under the auspices of the JRC Modelling Taskforce. The JRC-EU-TIMES model was developed as an evolution of the Pan European TIMES (PET) model of the RES2020 project, followed up within the REALISEGRID and REACCESS European research projects. The detailed residential, services and hydrogen modules and database of the JRC-EU-TIMES have been incorporated with additional modifications to MIRET-EU. Therefore, the modelling framework of MIRET-EU follows the same framework developed successively in the PET36, the JRC-EU-TIMES, MIRET-FR (French version developed by IFPEN since 2010) and TIAM-IFPEN (the world version being developed by IFPEN since 2019) models with additional expertise from IFP Energies Nouvelles in specific sectors such as transport, refineries and bioenergy conversion technologies, hydrogen infrastructure, power sector and industry.

MIRET-EU encompasses all stages from primary resources through the chain of processes that transform, transport, distribute and convert energy into the supply of energy services demanded by energy consumers. On the energy supply side, it comprises fuel production, primary and secondary energy sources, and imports and exports. Through various energy carriers, energy is supplied to the demand side, which is structured into residential, commercial, agricultural, transport and industrial sectors (Supplementary Figure 2).

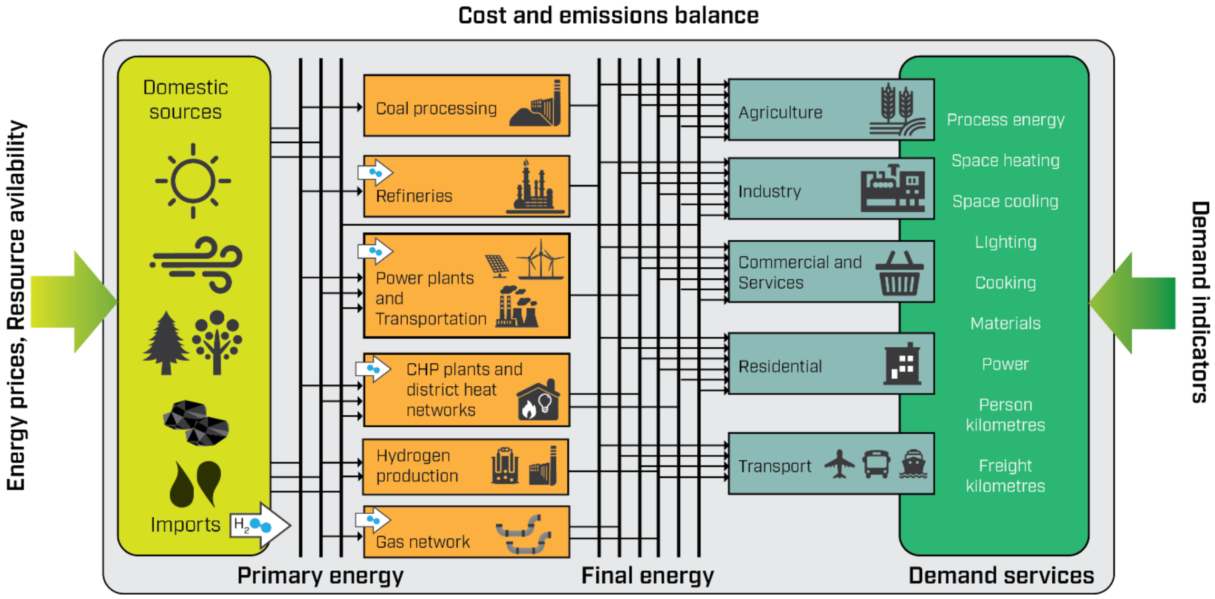

Supplementary Figure 2. Partial view of the Reference Energy System with its interdependencies in MIRET-EU model[3].

The reference energy economy is thus composed (from left to right) of:

- A primary energy supply block, which includes imported primary energy sources (uranium, crude oil, coal, natural gas); biomass which has been disaggregated into four types of commodity groups to better consider the competition between their consumption in biofuels production (1st and 2nd generation), hydrogen production, power sector, industry, residential and commercial. These groups are derived from the JRC database ENSPRESO (Energy System Potentials for Renewable Energy Sources) related to bioenergy potentials for EU and neighbouring countries[4]. Agriculture, forestry, and waste are the main sectors providing biogenic resources for energy production following the ENSPRESO database. The biomass resources have been disaggregated into sugar beet, starch, rape-seed and lignocellulosic potentials, municipal waste, and industrial waste-sludge potentials, and biogas potentials provided by dry and wet manure coming from cattle. In addition to the resource potentials, the related supply costs have also been provided in ENSPRESO to determine the systemic impact of biomass, together with other system-related variables, such as carbon price[4]; and imported raw materials for industry sectors.
- An energy technology block, whose technologies transform primary energy into energy vectors and energy services. It includes the electricity generation (all power plants from fossil-based to renewable energy sources). Oil refining and biofuel units are modelled based on IFPEN's approach and recognized expertise in the field of refineries and biofuels. The production chain is divided into feedstock pre-processing, production processes, and blending (blending of diesel B7, B10), gasoline SP95 grades E5, E10 and E85, and jet fuels). First generation biofuel production is subdivided into four sources: ethanol production from sugar beet and starch feedstock's, the trans-esterification and hydro-treatment of crushed oilseeds into FAME (Fatty Acid Methyl Esters) and HVO (Hydro treated Vegetable Oils), respectively. Second generation

production is subdivided into two sources: ethanol and synthetic FT-Diesel from lignocellulosic feedstocks. The end-use technologies are related to agriculture, industry, transport, residential and services (see below for more sectorial details).

- A final energy / energy services demand block such as industrial demands, space and water heating demands, mobility demands in the transport sector, trades (oil products, electricity, hydrogen, CO_2 captured), etc.
- A policy block which includes measures and constraints of several types affecting all sectors. Some are of microscopic nature, such as quality norms for refinery products, the number of functioning hours of fuel turbines, power plants, etc. Some are macroscopic in nature, e.g., global emission constraints or sectoral restrictions.

Strengths and weaknesses of the MIRET-EU model

MIRET-EU is an economic model with a rich technology representation for estimating capacity investment pathways over the long term. It combines two different, but complementary, systematic approaches to energy system modelling: a technical engineering approach and an economic approach. TIMES uses linear programming to produce a least-cost energy system, optimised across regions and sectors according to several user constraints, over medium to long-term time horizons. This unique objective function guarantees the internal consistency of the resulting scenario, as the decision criteria are the same for all processes and flows. These types of models are effective for assessing long-term investment decisions in complex systems where future technologies are different from current technologies.

The TIMES model assumes perfect foresight over the entire horizon, i.e., all investment decisions are made in each period with full knowledge of future events. This technology-detailed model provides insights to decision-makers regarding energy systems to determine which technologies are competitive, marginal, or uncompetitive in each market according to dynamic economic cost-benefit analyses. In short, MIRET is used for the exploration of possible energy futures based on contrasted scenarios.

As a partial equilibrium model, MIRET-EU does not model economic interactions outside the European energy sector.

The model is well suited to the development of Energy Roadmaps by making explicit the representation of technologies and fuels in all sectors to anticipate achievable futures based on actual knowledge. This is relevant for investment decisions in complex systems with differences between existing and future technologies. The model optimizes operation and investment decisions based on the characteristics of alternative generation technologies, energy supply economics, and environmental criteria. TIMES is thus a vertically integrated model of the entire extended energy system. The modelling uses exogenous cost data, rather than inclusion of the trade-off between the installed capacities and the technology cost reduction (learning-by-doing). The scope of the model extends beyond purely energy-oriented issues, to include the representation of environmental emissions, and materials, related to the energy system. In addition, the model is suitable for the analysis of energy-environmental policies,

which may be accurately represented by making explicit the representation of technologies and fuels in all sectors. The great flexibility of TIMES, especially at the technological level, allows the representation of almost all policies, whether at the national, sectorial, or sub-sectorial level. Finally, the model is driven by explicit exogenous final energy services demand and fuel prices.

On the other hand, some limitations inherent to this type of model can be pinpointed. MIRET-EU is data consuming; therefore, data availability could limit the scope and depth of possible analyses. Moreover, there is no explicit representation of macro-economic factors which means there are no feed-back loops between the effects of energy system changes and the economy (but they could be considered exogenously through the price elasticities of service demand). As all models are simplified representations of reality and its complex dynamics, they inherently have limitations as to the detail and scope of their mathematical representation. These simplifications, e.g., time and spatial resolution, sector or technology representation and system boundaries, which are mostly due to the data availability, may represent significant modelling limitations. Long computational times could be observed due to a very detailed representation of the complex energy system. The model is also sensitive to the data assumptions for emerging technologies, which are more uncertain and decision makers in practice do not always balance efforts across regions and sectors. Decision making that conditions investment in new technologies is often not economically rational, however representing non-rational decisions could be done via exogenous constraints. This does not allow capturing in detail all the aspects related to consumer behaviour, which play a fundamental role in decision-making processes. Even if the decision making is rational[5], it is often not based on least-cost criteria. Policy rationality may stress effectiveness, equity issues, timing, risk, and other factors that are not accounted for in this framework. The optimistic view of the future due to the perfect foresight approach which does not account for real-world uncertainty. However, it is possible to implement via the model to have foresight over a limited part of the horizon, such as one or a few periods or to temper it by using higher discount rates. By so doing, a modeler may attempt to simulate real-world decision-making conditions, rather than socially optimal ones[1]. In this article, there is no disaggregation by plant size unlike in the MIRET-FR model (France) due to a lack of data and the consequences of so doing on computational time. This implies, as a simplification, that all installations in industry and CHP are considered as falling within the scope of the EU ETS Directive. The reconciliation of the very short-term physical dynamics (e.g., integrating system adequacy, transient stability analysis in the power sector) into long-term prospective models is not considered in this multiregional model MIRET-EU.

Sectoral representation

MIRET-EU provides a disaggregated representation of energy demand. In this section, the different energy end-use sectors are described in detail for a better comprehension of the sectorial assumptions/modelling. MIRET-EU carries out its optimization horizontally across all sectors and vertically across all technologies delivering the same commodity, regions, and time periods for which the limit is imposed. Supplementary Figure 3 shows how substitutions are considered in the model within the different sectors.**Error! Reference source not found.**

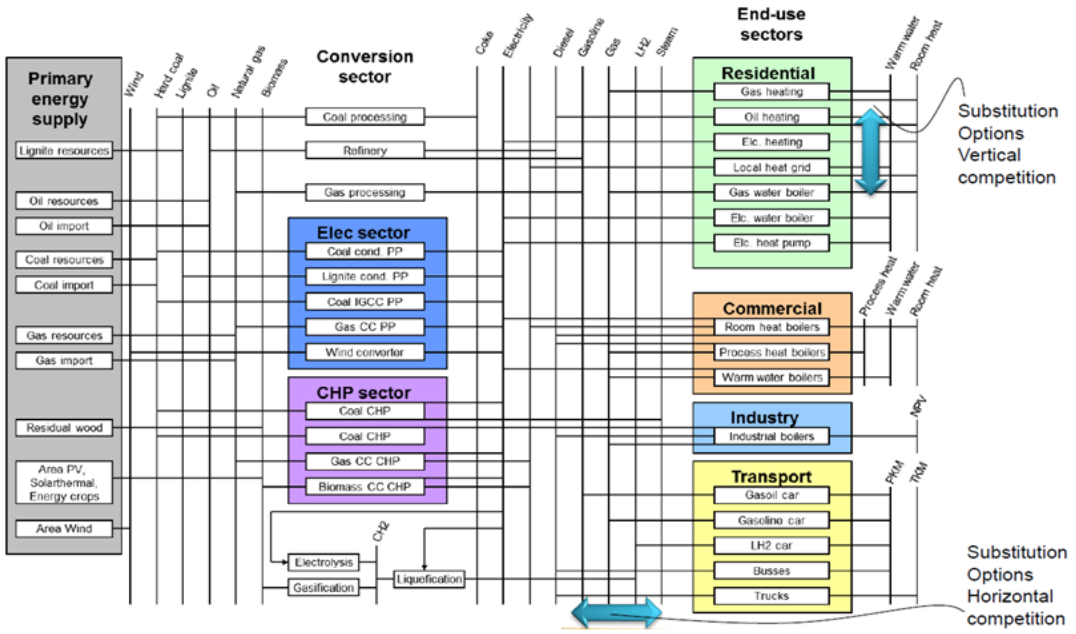

Supplementary Figure 3. Substitution options in MIRET-EU[6]

As above-mentioned, MIRET-EU's reference energy system encompasses all the steps from primary resources through the chain of processes that transform, transport, distribute and convert energy into the supply of energy services demanded by energy consumers. The details of each sector represented in the model are presented below. Throughout each description, modelling competencies are identified and limits, which are generally due to the lack of available database, are pointed out. The model considers the existing technologies which are related to what is already installed in all considered countries in the historical period (i.e., the 2010s period), and the new technologies which are to be available in the future (e.g., from 2020 onwards).

Power sector. This sector can be divided in five parts (Supplementary Figure 4): primary resources, power plants, power grid, demand (end-uses) and emissions. The primary resource's part is disaggregated into import and mining processes to also consider domestic extraction of resources in each country. These primary resources are converted into other energy carriers (electricity, heat, refinery products) via heat and power plants, cogeneration heat plants (CHP) or refinery plants. The power grid is explicitly represented in MIRET-EU in a simple manner to consider the different voltage levels. All these energy carriers are consumed in the end-uses/processes in agriculture (machine drives, heat uses, etc.), industry (iron & steel, cement, pulp & paper, etc.), transport (cars, buses, etc.), residential and services (space heating, cooling, cooking, lighting, etc.) sectors. Balancing the demand in all later sectors (mobility, tonnes of cement, space heating) could imply CO_2 emissions as in energy conversion technologies. The CO_2 emissions could come from all demand and supply sectors (each energy carrier has an associated emission factor) and could be released to the atmosphere or captured via carbon capture and storage (CCS). CCS is considered in some industrial processes (ammonia, iron & steel, cement, etc.) and in power generation and supply sectors (hydrogen and biofuel production). The model includes CO_2 from carbon capture or from the atmosphere directly by

using direct air capture (DAC). The captured emissions can be from fossil or biogenic sources. Afterwards, they are either stored permanently in sinks, traded, or reused (CCUS routes) to produce synthetic fuels (PtL), or methane (PtM) which could be incorporated in the natural gas grid. The techno-economic details of electricity generating technologies rely on updated power generation technology assumptions (efficiency, capital costs, fixed and variable O&M costs) from the JRC database released in October 2019 by the European Commission, as well as from the IEA database. An evolution of these technology characteristics is provided up to 2050. Several country-specific assumptions are introduced in the model such as short and/or medium-term expected phase out and roll out of technologies, etc.

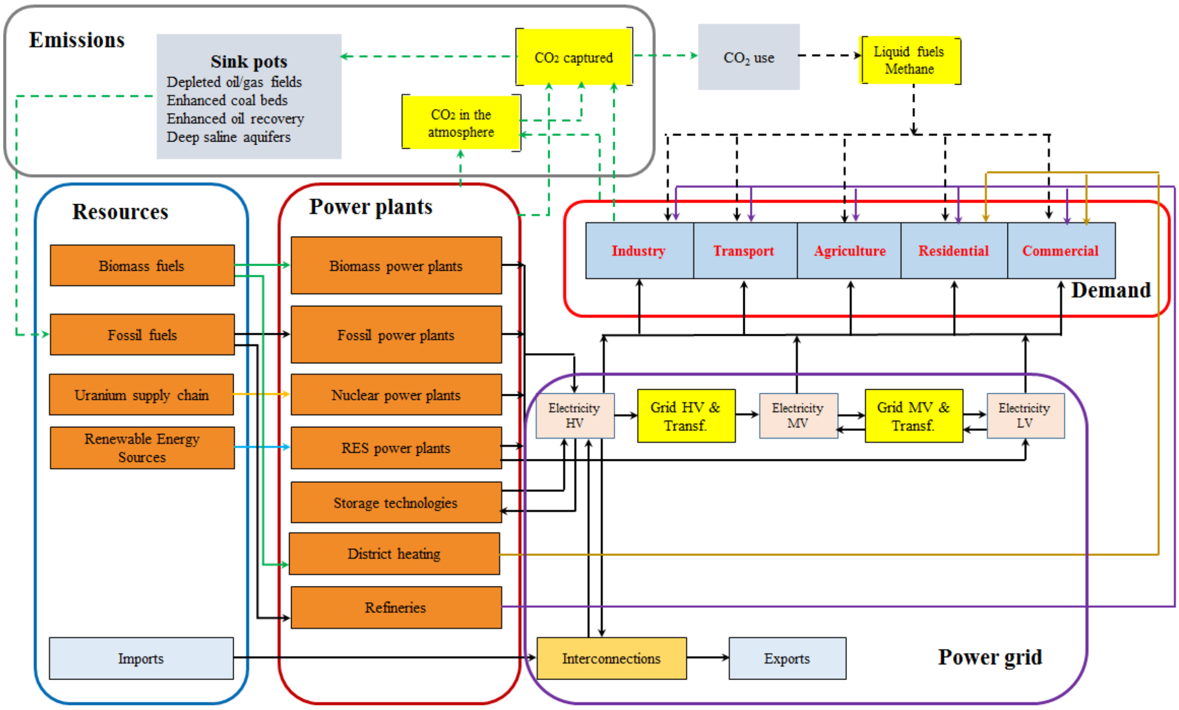

Supplementary Figure 4. Power sector representation in MIRET-EU

Residential, Commercial and Agriculture sectors. The needed data of the residential, services and agriculture sectors have been taken from the JRC-EU-TIMES database with a very disaggregated representation. MIRET-EU considers space heating, space cooling, water heating, cooking, ventilation, ICT and multimedia, and other electric appliances as end-uses in the residential and commercial sectors, while a single energy service demand satisfied by a single technology that consumes a mixture of fuels via different end-uses is considered for the agriculture sector. The JRC-IDEES (Integrated Database of the European Energy System), released in July 2018, provides very detailed information on the energy system and its underlying drivers for all EU Member States in annual time steps starting from the year 2000 up to 2015 consistent with Eurostat statistics in the last 2018-version. The model calibration has been continued to 2020 when other existing data between 2015-2020 are available. JRC-IDEES has been very useful to calibrate the historical evolution of the energy sector in MIRET-EU. The details of the end-use technologies (boilers, heat-pumps, CHP, district heating, water heaters, among others) for residential and services are based on a wide literature review,

including, the technology pathways described by the EU-funded project Advanced System Studies for Energy Transition, the ENTRANZE database, the Eco-design requirement reports of the European Commission, PRIMES data, VHK reports, among others.

Industry sector. The modelling framework of the industry is divided into two categories according to previous work and studies: manufacturing process of the energy intensive industries were described by their associated energy consumption ratios for each product. Choices are possible between several alternative process solutions[7], while the non-energy intensive industries are modelled by energy end-uses (mechanical processes, heat treatment, evaporation, and concentration, drying, etc.) due to the unsuitability of the product/process approach[8]. The BREFs, which are the most complete series of reference documents covering industrial activities, provide descriptions of a range of industrial processes and, for example, their respective operating conditions and emission rates. It should be noted that CCS is considered in some energy intensive industries such as cement, glass, pulp & paper, ammonia, iron & steel, etc. The calibration of MIRET-EU relies on the JRC-IDEES, EUROSTAT, IEA database and on the framework of the JRC-EU-TIMES and TIAM-IFPEN.

Transport sector. The transport sector in MIRET-EU is based on IFPEN previous transport structure of MIRET-FR and TIAM models for critical raw material analyses in this sector up to 2050[9]. It is subdivided into four modes: road, rail, navigation, and aviation (Supplementary Figure 4).

The road transport has been divided into passenger light-duty vehicles (PLDV) (small, medium and large), buses, commercial vehicles (CV - light, heavy and medium trucks) and 2/3-wheelers. The presentation of technologies relies on a specific understanding of the transport sector within each segment (PLDV, CV, bus, minibus, and 2/3-wheelers). The existing and future vehicles have been implemented with their techno-economical parameters - fuel efficiency, average annual vehicle mileage, lifespan, cost (purchase cost, fixed and variable operation and maintenance costs), etc. All these attributes have been derived from the IEA Mobility Model (IEA MoMo) data on transport[10] and the JRC database.

For rail, MIRET-EU considered the non-urban rail, urban rail, and freight rail while for the aviation and navigation, they have been disaggregated into freight and passengers, inland and bunkers. Contrary to MIRET-FR where all existing and future different aircrafts have been considered to allow alternative technologies, it has been assumed to consider single generic technologies at the European level with an average efficiency to satisfy the aviation activity, likewise for navigation. In addition, ammonia for navigation and pure hydrogen for aviation are not considered in the model for the moment.

The potential role of ammonia in the energy system has been the subject of many discussions, particularly as a marine fuel. However, as ammonia has been represented as an industrial sector and not as a feedstock, it has been proposed a simplified approach which add hydrogen in the fuel mix of a generic ship on an equivalent basis. Thus, the hydrogen demand gives some indications of the potential demand of ammonia as a shipping fuel until 2050. Transport fuels

considered in the model are hydrogen, synthetic fuels (XtL), e-fuels (PtL), natural gas, liquefied petroleum gas (LPG), blending of gasoline, diesel, and jet fuels.

The production chain of biofuels is divided into feedstock pre-processing (where vegetable oil, starch grain, sugar beet and lignocellulosic are pre-processed), and production processes which are grouped into first- and second-generation biofuel processes.

Representation of the hydrogen supply chain. Regarding the hydrogen supply chain structure, the production options are disaggregated under centralised vs. decentralised and by size (large, medium and/or small), as presented in Supplementary Figure 5.

In the decentralised option, hydrogen is produced close to where it is consumed, whereas in the centralised option, large scale hydrogen facilities are considered producing hydrogen that needs to be delivered to end-users via an extensive transport and distribution infrastructure. Most of the hydrogen techno-economic assumptions considered in the model have been provided by the JRC to IFP Energies Nouvelles in June 2019. They are based on the JRC hydrogen structure in TIMES input data available for the development of the hydrogen sector in MIRET[11-17].

In total, more than 30 hydrogen production options are considered by process type (with and without CCS), by size, by system design (centralised vs decentralised): hydrogen from electrolysis, hydrogen from fossil fuels, natural gas steam reforming with and without CCS, auto-thermal reformer (ATR)/ gas-heated reformer (GHR) with CCS, methane pyrolysis, partial oxidation of heavy oil, coal gasification with and without CCS, hydrogen from biomass, biomass Gasification with and without CCS, and ethanol steam reforming.

A data request regarding the techno-economic assumptions on hydrogen production technologies considered in the MIRET-EU model has been extensively discussed with technical experts to cross validate and complete the list. The implementations were made based on the data provided.

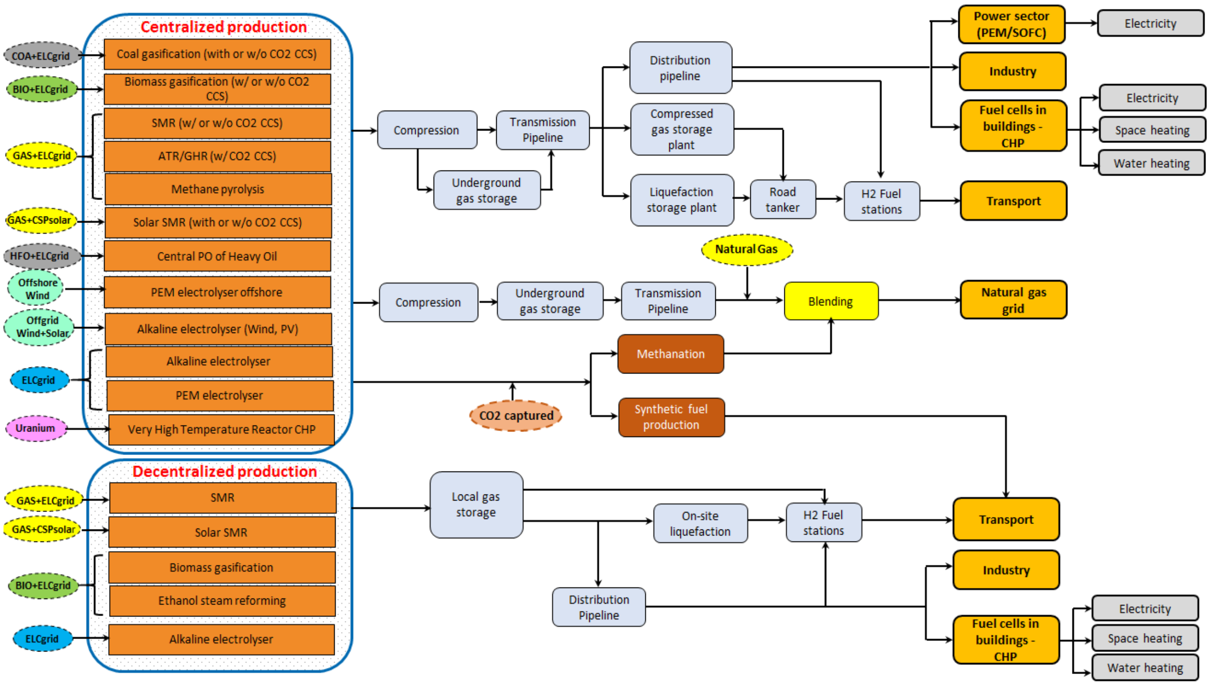

Supplementary Figure 5. Hydrogen supply chain in MIRET-EU[2]

Thus, different input energy sources for hydrogen (e.g., electricity from grid, PV, wind, etc.) have been considered in the model. The JRC representation of the hydrogen supply chain has been improved by adding new hydrogen production options such as off-grid wind and PV with electrolysers, methane pyrolysis, autothermal reformer (ATR)/gas-heated reformer (GHR).

Hydrogen delivery begins with hydrogen conditioning and is completed with supplying hydrogen to end users. Hydrogen delivery is modelled by creating aggregated processes coupling several hydrogen delivery sub processes. Consequently, an aggregated delivery process is formed by summing all processes of a probable hydrogen value chain, from conditioning to immediately before end-use application[16]. Total costs for each of the delivery path result from the cost aggregation of the individual steps. Depending on the selected pathway of hydrogen delivery, sub processes include hydrogen storage options (e.g., underground salt caverns, liquid storage bulk, gas storage bulk and local gas storage bulk), liquefaction, compression, distribution pipeline, road transportation and refuelling stations, liquid to liquid, liquid to gas, and gas to gas with small capacity (300 kg/day) and large capacity (1200 kg/day).

Regarding end-use applications of hydrogen, hydrogen gas, as a transport commodity can be consumed in buses, cars and commercial vehicles, and marine bunkers. The hydrogen use in trains and aviation is not yet included so far in the model, however the use in trains could be implemented in the future according to existing and available data as plans involving hydrogen trains already exist in several countries (two hydrogen trains in Germany)[17]. In the residential and commercial sectors (space heating, water heating and electricity via fuel cells-CHP), and for industrial processes, hydrogen gas and hydrogen-natural gas blending are also possible. For the blending with natural gas, within the current natural gas infrastructure, a maximum of 5% until 2025, 10% from 2025 and 15% from 2030 onwards has been assumed in MIRET-EU to

be in line with the hypothesis considered in the global TIAM IFPEN model, IEA-ETSAP TIAM version, or other European TIMES versions. According to the IEA[17], it is 10% blending max with 8% allowable under certain circumstances in Germany, while it is around 6% in France, 5% in Spain, 4% in Austria, and under 2% in other European countries. The Ameland project in the Netherlands did not find any problem for household devices to blend hydrogen up to 30%.

Hydrogen and natural gas separation is not considered in the model. In the power sector, proton exchange membrane (PEM) fuel cell technology is represented in the model to consider the penetration of hydrogen in power generation. Hydrogen can also be consumed in biorefineries and within carbon capture and utilisation (CCU) routes for Power-to-Liquid (PtL), and Power-to-Methane (PtM).

The hydrogen production technologies and the related cost and efficiency data are given in the following tables (Supplementary Tables 1 and 2).

Supplementary Table 1. Hydrogen production technologies – Cost data. All cost data is provided in the lower heating value (LHV)[11-15,18-24]

**Technology**	**Investment costs [€/kWH$_2$]**			**Fixed O&M [€/kWH$_2$]**			**Variable O&M [€/GJ]**			**Source**
	2020	2030	2050	2020	2030	2050	2020	2030	2050	
**Coal gasification, large size, centralised**	2363	2363	2363	118	118	118	0.16	0.12	0.12	6, 9, 10
**Coal gasification, medium size, centralised**	2929	2929	2929	147	147	147	0.22	0.22	0.22	6, 9, 10
**Coal gasification + CO_2 capture, large size, centralised**	2460	2460	2460	123	123	123	0.26	0.26	0.26	6, 9, 10
**Coal gasification + CO_2 capture, medium size, centralised**	3376	3376	3376	169	169	169	0.26	0.26	0.26	6, 9, 10
**Biomass gasification, small size, decentralised**	3099	3099	3099	81.9	81.9	81.9	1.3	1.3	1.3	6, 8, 9, 10
**Biomass gasification, medium size, centralised**	2959	2929	2929	146	146	146	0.45	0.45	0.45	6, 8, 9, 10
**Biomass gasification + CO_2 capture, medium size, centralised**	3376	3376	3376	169	169	169	0.46	0.46	0.46	6, 8, 9
**SMR, large size, centralised**	805	805	805	37.8	37.8	37.8	0.08	0.05	0.05	1, 9
**SMR, medium size, decentralised**	1945	1509	1509	52.7	29.9	29.9	0.04	0.04	0.04	6, 8, 10
**SMR + CO_2 capture, large size, centralised**	1487	1204	1133	44.6	36.1	34.0	0.53	0.07	0.07	1, 9
**ATR + CO_2 capture, large size, centralised**	800	700	700	24.0	21.0	21.0	0.53*	0.07***Error! Bookmark not defined.**	0.07*	8, 9

**GHR + ATR + CO_2 capture, large size, centralised**	830	750	750	24.9	22.5	22.5	0.53*	0.07*	0.07*	8, 9
**Ethanol steam reforming, decentralised**	2700	2700	2700	0	0	0	19.65	19.65	19.65	6
**PEM electrolyser**	1750	795	476	53	46	46	0.15**	0.06**	0.06**	2, 3, 7, 9, 10
**Alkaline electrolyser, large size, centralised**	1250	576	345	19	15	15	0.15	0.06	0.06	2, 3, 7, 9, 10
**Alkaline electrolyser, wind off grid, centralised**	2408	1807	1493	59	52	50	0.15	0.06	0.06	2, 3, 7, 9, 10
**Alkaline electrolyser, PV off grid, centralised**	1878	1083	707	37	27	24	0.15	0.06	0.06	2, 3, 7, 9, 10
**PEM electrolyser, offshore, centralised**	4535	3245	2709	115	95	90	0.96	0.17	0.17	2, 3, 7, 9, 10
**Alkaline electrolyser, small size, decentralised**	1250	576	345	19	15.0	15	0.96	0.17	0.17	2, 3, 7, 9, 10
**Very High Temperature Reactor CHP, centralised**	NA	4687	3937	NA	304.7	255.9	NA	2.60	2.60	6, 10
**Methane pyrolysis (Kvaerner process), centralised**	1993	1993	1993	89.8	89.8	89.8	0.70	0.70	0.70	6
**Molten media methane pyrolysis, large size**	778	778	778	64.9	64.9	64.9	-	-	-	11
**Non-catalytic methane pyrolysis, small size**	1751	1751	1751	225	225	225	-	-	-	12

* The variable O&M costs for GHR + ATR and ATR are based on the reported values for SMR + CO_2 capture as they are mostly related to process water, cooling water, and catalyst replacement.
** The variable O&M costs for PEM electrolyser are based on Alkaline electrolyser, large size as they are mostly related to process and cooling water.

Supplementary Table 2. Hydrogen production technologies – Technology Data[11-15,18-24]

Technology	Size [MW]	Fuel Efficiency [PJ/PJ$_{H2}$] (LHV)				Life			Source
		Fuel	2020	2030	2050	2020	2030	2050	
**Coal gasification, large size, centralised**	1667	Coal	1.67	1.67	1.67	25	25	25	6, 9, 10
**Coal gasification, medium size, centralised**	434	Coal	1.67	1.67	1.67	25	25	25	6, 9, 10
**Coal gasification + CO_2 capture, large size, centralised**	1667	Coal	1.72	1.72	1.72	25	25	25	6, 9, 10
**Coal gasification + CO_2 capture, medium size, centralised**	442	Coal	1.72	1.72	1.72	25	25	25	6, 9, 10
**Biomass gasification, small size, decentralised**	0.7	Biomass	2.10	2.10	2.10	25	25	25	6, 8, 9, 10
		Grid electricity	0.03	0.03	0.03				
**Biomass gasification, medium size, centralised**	33	Biomass	2.10	2.10	2.10	25	25	25	6, 8, 9, 10
		Grid electricity	0.03	0.03	0.03				
**Biomass gasification + CO_2 capture, medium size, centralised**	33	Biomass	2.10	2.10	2.10	25	25	25	6, 8, 9
		Grid electricity	0.03	0.03	0.03				
**SMR, large size, centralised**	1530	Natural gas	1.32	1.32	1.32	25	25	25	1, 9
		Grid electricity	-0.02	-0.02	-0.02				

**SMR, medium size, decentralised**	2	Natural gas	1.36	1.27	1.27	25	25	25	6, 8, 10
		Grid electricity	0.25	0.07	0.07				
**SMR + CO_2 capture, large size, centralised**	1502	Natural gas	1.385	1.385	1.385	25	25	25	1, 8, 9
		Grid electricity	0.015	0.015	0.015				
**ATR + CO_2 capture, large size, centralised**	1260	Natural gas	1.36	1.36	1.36	25	25	25	8, 9
		Grid electricity	0.04	0.04	0.04				
**GHR + ATR + CO_2 capture, large size, centralised**	1260	Natural gas	1.28	1.20	1.20	25	25	25	8, 9
		Grid electricity	0.06	0.05	0.05				
**Ethanol steam reforming, decentralised**	0.01	Ethanol	1.47	1.47	1.47	10	10	10	6
		Grid electricity	0.08	0.08	0.08				
**PEM electrolyser**	NA*	Grid electricity	1.60	1.55	1.55	6**	7	9	2, 3, 7, 9, 10
**Alkaline electrolyser, large size, centralised**	72	Grid electricity	1.55	1.45	1.45	20	20	20	2, 3, 7, 9, 10
**Alkaline electrolyser, wind off grid, centralised**	NA*	Wind off grid	1.55	1.45	1.45	30	30	30	2, 3, 7, 9, 10
**Alkaline electrolyser, PV off grid, centralised**	NA*	PV off grid	1.55	1.45	1.45	30	30	30	2, 3, 7, 9, 10
**PEM electrolyser, offshore, centralised**	NA*	Wind offshore	1.5	1.5	1.5	20	20	20	2, 3, 7, 9, 10
**Alkaline electrolyser, small size, decentralised**	0,6	Grid electricity	1.55	1.45	1.45	20	20	20	2, 3, 7, 9, 10
**Very High Temperature Reactor CHP, centralised**	600	Uranium	NA	1.5	1.5	NA	60	60	6, 10
**Kvaerner process, centralised**	19	Natural gas	1.75	1.75	1.75	25	25	25	6
		Grid electricity	0.35	0.35	0.35				
**Molten media methane pyrolysis, large size**	420	Natural gas	2.05	2.05	2.05	20	20	20	11
**Non-catalytic methane pyrolysis, small size**	2.8	Natural gas	2.50	2.50	2.50	20	20	20	12

* No reference size for costs provided. However, it is expected that the sizes are in the range between *Alkaline electrolyser large size* and *Alkaline electrolyser small size*, that is between 0.6 MW and 72 MW.
** The lifetime in *PEM electrolyser* is increasing due to R&D. Direct application in offshore parks has a higher lifetime due to the lower capacity factor and may be limited by the lifetime of the offshore wind turbines.

Pipelines and trade representation in the MIRET-EU model

Natural gas and LNG. The trade of natural gas between European countries and other regions is modelled via a trade matrix that defines the existing and planned capacities until 2025 with the possibility of investing in additional capacity from 2025 onwards if needs be. The ENTSOG (European Network of Transmission System Operators for Gas) *Ten-Year Network Development Plan* (TYNDP) provides an overview of the European gas infrastructure and its future development[25]. It thus allows depicting the maximum existing and planned capacities to have bilateral or unilateral exchanges between European countries until 2025 (Supplementary Figure 6). Hereafter, an example of a matrix table which is used to declare the traded energy commodities (in this example, natural gas) and the links between countries (1=active links).

The countries at the left-most column represent the exporters and the ones at the top-most line are the importers. Two types of trade are considered in the model: either bilateral links between countries (e.g., trade between Germany - importer/exporter and Austria - importer/exporter) or unilateral links between countries (e.g., trade between Netherlands – importer and Norway - exporter).

Natural gas	Austria	Belgium	Bulgaria	Czech Rep.	Denmark	Estonia	Finland	France	Germany	Greece	Hungary	Ireland	Italy	Latvia	Lithuania	Netherlands	Poland	Portugal	Romania	Slovakia	Slovenia	Spain	Sweden	United Kingdom	Norway	Switzerland
Austria									1		1		1							1	1					
Belgium								1	1							1								1		
Bulgaria										1																
Czech Rep.									1											1						
Denmark									1							1							1			
Estonia																										
Finland																										
France		1																				1				1
Germany	1	1		1	1			1								1	1									1
Greece																										
Hungary																			1							
Ireland																								1		
Italy	1																			1	1					1
Latvia						1									1											
Lithuania														1												
Netherlands		1							1															1		
Poland																										
Portugal																						1				
Romania			1																							
Slovakia	1			1																						
Slovenia											1		1													
Spain								1										1								
Sweden																										
United Kingdom		1										1				1										
Norway		1						1	1							1								1		
Switzerland								1					1													

Supplementary Figure 6. Endogenous trade matrix of natural gas within European countries.

In addition to natural gas trade within European countries, natural gas trade from Russia (before 2022) and North Africa are also considered for Germany, Finland, Estonia, Lithuania, Hungary, Poland, Romania, Slovakia, Spain, and Italy (Supplementary Table 3).

Supplementary Table 3. Trade of natural gas from outside European countries

Natural gas Country origin	Natural gas Country destination
**Russia**	Germany
	Estonia
	Lithuania
	Hungary
	Poland
	Romania
	Slovak Republic
	Finland
	EU28
**North Africa**	Spain
	Italy

Supplementary Table 4. LNG import terminals considered.

LNG Terminals
Belgium
Croatia
Estonia
Finland
France
Germany
Greece
Ireland

Italy
Latvia
Lithuania
Netherlands
Norway
Poland
Portugal
Spain
Sweden
United Kingdom

Belgium, Croatia, Estonia, Finland, France, Germany, Greece, Ireland, Italy, Latvia, Lithuania, Netherlands, Norway, Poland, Portugal, Spain, and the UK have also the possibility to import LNG from outside Europe (Supplementary Table 4). The GIE (Gas Infrastructure Europe) LNG Map and the CEDIGAZ database provides comprehensive information on existing and under construction LNG Terminals in Europe. The project tables in the Annex A of the ENTSOG's TYNDP 2020 provides the planned or under study LNG terminals for the coming years with a detailed overview of their status. For the current study, only confirmed projects (in final investment decision) and those with advanced maturity status by end of 2021 have been considered.

Electricity. Electricity trade is represented like natural gas via a matrix table where the endogenous exchanges are represented. The ENTSO-E (European Network of Transmission System Operators for Electricity) *Ten-Year Network Development Plan* (TYNDP) also provides an overview of the maximum existing and planned capacities until 2030[25]. The model also considers a maximum level of imports and exports of electricity from outside Europe.

Hydrogen transport. Regarding hydrogen transport, if a reduced demand for gas is observed, capacity could become available and could be used to transport hydrogen by repurposing segments of the natural gas pipelines to hydrogen, or by investing in new hydrogen pipelines[26]. Therefore, two types of pipelines for hydrogen trade have been modelled in MIRET-EU: retrofitted natural gas pipelines to hydrogen and new dedicated hydrogen pipelines. Therefore, the model optimises between retrofitting existing natural gas infrastructure for hydrogen carrying capability on one hand, and alternatively, or additionally, investing in new dedicated hydrogen infrastructure on the other hand[27]. Thus, the retrofitted gas pipelines to hydrogen have been assumed to have the same matrix between the countries as the existing gas pipelines. The possibility of investing in additional hydrogen transport in the future is also considered within the existing gas matrix[20,28]. An estimate of the investment and operating costs have been provided in the 2020 *European Hydrogen Backbone* report[29] for new and refurbished pipelines dedicated to hydrogen (Supplementary Table 5).

Supplementary Table 5. Cost input ranges used for estimating total investment, operating, and maintenance costs for hydrogen infrastructure. Values are for 48-inch pipelines (one of the widest pipeline types in the intra-EU gas network)[29].

Cost element	Unit	Low	Medium	High
**Pipeline CAPEX new**	M€/km	2.5	2.75	3.36
**Pipeline CAPEX retrofit**	M€/km	0.25	0.5	0.64
**Compressor station CAPEX**	M€/MWe	2.2	3.4	6.7

**Operating & maintenance costs**	€/year as a % of CAPEX		0.8-1.7%	

The transport modalities considered are maritime transport (LH_2 and ammonia) and cross-border interconnections (subsea or aboveground). Hydrogen supply curves (based on levelized cost of hydrogen - LCOH - trajectories) from extra-European countries are provided by a specific study carried out (the HyPE model). The latter is included in the model as an alternative for hydrogen supply, competing with domestic production within Europe. The maximum import volumes are based on the maximum possible trade flows between the country of origin and the entry point within Europe, with references to transport costs and constraints on planned infrastructure.

CO_2 flows and CCUS routes in the MIRET-EU model. The model recovers CO_2 from carbon captured in industry, electricity and hydrogen and biofuel production sectors or from the atmosphere directly via DAC (Supplementary Figure 7). The captured emissions can be from fossil or biogenic sources. Afterwards, they are either stored permanently in sinks (depleted oil/gas fields, enhanced coal beds, enhanced oil recovery, deep saline aquifers), traded or reused to produce synthetic fuels (PtL – power-to-liquid), or methane (PtM – power-to-methane). The main sources of data are Blanco et al.[11-12] and Meylan et al.[30]. Regarding CO_2 transport considered in the model [16, 31-33], the pipeline trade among European countries is modelled via a trade matrix that defines the links between regions. CO_2 transport by tanker could also be implemented provided cost data is available (e.g., France-Norway planned CO_2 trade by tanker).

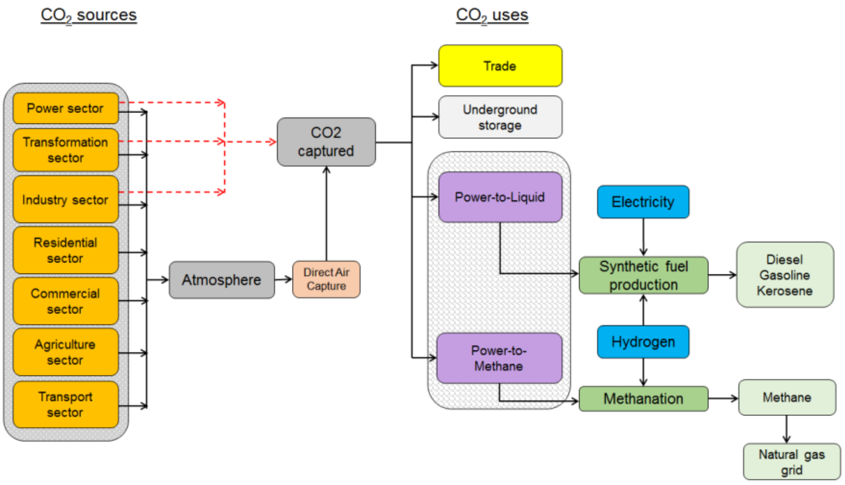

Supplementary Figure 7. CCUS routes in the MIRET-EU (Transformation sector encompasses biofuel and hydrogen production).

Energy policy assumptions in MIRET-EU. In MIRET-EU, the modelling framework is aligned with the agenda of the European Green Deal and EU pillars and targets, incorporating the policy assumptions both at the country and EU-level explicitly represented through constraints. They include sectors covered by the overall EU emissions targets for 2030 and 2050, the targets for the sectors covered by the EU ETS and that of non-EU ETS sectors, the

Energy Efficiency Directive with particular attention to the transport sector, the Renewable Energy Directives and the NECPs as detailed in *Hydrogen for Europe* study[2].

Supplementary References

1. Loulou, R., Goldstein, G., Kanudia, A., Lehtila, A., & Remme, U. Documentation for the TIMES model, energy technology systems analysis program (ETSAP) of the international energy agency (2016).

2. Hydrogen 4EU. Charting pathways to enable net zero – 2022 edition (2022). https://www.hydrogen4eu.com/.

3. Remme, U., & Mäkela, J. *TIMES training Workshop*. Gothenburg (2001).

4. Ruiz, P., Nijs, W., Tarvydas, D., Sgobbi, A., Zucker, A., Pilli, R., Jonsson, R., Camia, A., Thiel, C., Hoyer-Klick, C. and Dalla Longa, F. ENSPRESO-an open, EU-28 wide, transparent and coherent database of wind, solar and biomass energy potentials. *Energy Strategy Reviews*, *26*, p.100379 (2019).

5. Gielen, D., Taylor, M. Modelling industrial energy use: The IEAs Energy Technology Perspectives, *Energy Economics 29* (4), 889-912 (2007).

6. Gargiulo M. Introduction to the TIMES model generator. *TIMES Training Workshop*, Paris, France (2018).

7. Djemaa, A. *Modélisation bottom-up, un outil d'aide à la décision long terme pour les mesures politiques en matière d'énergie et d'environnement: le modèle TIMES appliqué aux industries grandes consommatrices d'énergie* (Doctoral dissertation, École Nationale Supérieure des Mines de Paris, 2009).

8. Seck, G. S., Guerassimoff, G., & Maïzi, N. Heat recovery with heat pumps in non-energy intensive industry: A detailed bottom-up model analysis in the French food & drink industry. *Applied Energy*, *111*, 489-504 (2013).

9. Hache, E., Seck, G. S., Simoen, M., Bonnet, C., & Carcanague, S. Critical raw materials and transportation sector electrification: A detailed bottom-up analysis in world transport. *Applied Energy*, *240*, 6-25 (2019).

10. Fulton, L., Cazzola, P., & Cuenot, F. IEA Mobility Model (MoMo) and its use in the ETP 2008. *Energy Policy*, *37*(10), 3758-3768 (2009).

11. Blanco, H., Nijs, W., Ruf, J., & Faaij, A. Potential for hydrogen and Power-to-Liquid in a low-carbon EU energy system using cost optimization. *Applied energy*, *232*, 617-639 (2018).

12. Blanco, H., Nijs, W., Ruf, J., & Faaij, A. Potential for hydrogen and Power-to-Liquid in a low-carbon EU energy system using cost optimization. *Applied energy*, *232*, 617-639 (2018).

13. Bolat, P., & Thiel, C. Hydrogen supply chain architecture for bottom-up energy systems models. Part 1: Developing pathways. *International journal of hydrogen energy*, *39*(17), 8881-8897 (2014).

14. Bolat, P., & Thiel, C. Hydrogen supply chain architecture for bottom-up energy systems models. Part 2: Techno-economic inputs for hydrogen production pathways. *International journal of hydrogen energy*, *39*(17), 8898-8925 (2014).

15. Sgobbi, A., Nijs, W., De Miglio, R., Chiodi, A., Gargiulo, M., & Thiel, C. How far away is hydrogen? Its role in the medium and long-term decarbonisation of the European energy system. *international journal of hydrogen energy*, *41*(1), 19-35 (2016).

16. Simoes, S., Nijs, W., Ruiz, P., Sgobbi, A., Radu, D., Bolat, P., Thiel, C., & Peteves, E. *The JRC-EU-TIMES model-assessing the long-term role of the set plan energy technologies* (2013).

17. Krewitt, W., & Schmid, S. Fuel cell technologies and hydrogen production/distribution options. *EU-Project CASCADE Mints.* Deutsches Zentrum fur Luft-und Raumfahrt, Stuttgart (2005).

18. IEA. *The Future of Hydrogen*. IEA (2019). https://www.iea.org/fuels-and-technologies/hydrogen.

19. Schmidt O., Gambhir A., Staffel, I., Hawkes, A., Nelson, J., Few, S. Future cost and performance of water electrolysis. *International Journal of Hydrogen Energy 42*, 30470-30492 (2017).

20. H21NoE *H21 NoE Report*. England (2018).

21. Ramsden, T., Steward, D. and Zuboy, J. *Analyzing the levelized cost of centralized and distributed hydrogen production using the H2A production Model version 2*, NREL Technical Report TP-560-46267 (2009).

22. Ramsden, T., Ruth, M., Diakov, V., Laffen, M., Timbario, T.A. *Updated Cost, Well-to-Wheels Energy Use, and Emissions for the Current Technology Status of Ten Hydrogen Production, Delivery, and Distribution Scenarios*. NREL Technical Report NREL/TP-6A10-60528 (2013).

23. Parkinson, B., Tabatabaei, M., Upham, D. C., Ballinger, B., Greig, C., Smart, S., McFarland, E. Hydrogen production using methane: Techno-economics of decarbonizing fuels and chemicals. *International Journal of Hydrogen Energy 43*, 2540-2555 (2018).

24. Keipi, T., Tolvanen, H., Konttinen, J. Economic analysis of hydrogen production by methane thermal decomposition: Comparison to competing technologies. *Energy Conversion and Management 159*, 264-273 (2018).

25. ENTSO-E & ENTSO-G. TYNDP 2020: Joint Scenario Report (2020).

26. Peters, D., van der Leun, K., Terlouw, W., van Tilburg, J., Berg, T., Schimmel, M., van der Hoorn, I., Buseman, M., Staats, M., Schenkel, M. and Mir, G.U.R. *Gas decarbonisation pathways 2020–2050: gas for climate* (2020).

27. De Vita, A., Kielichowska, I., Mandatowa, P., Capros, P., Dimopoulou, E., Evangelopoulou, S., Fotiou, T., Kannavou, M., Siskos, P., Zazias, G. and De Vos, L. *Technology pathways in decarbonisation scenarios.* Tractebel, Ecofys, E3-Modelling. Brussels, Belgium (2018).

28. Schoots, K., Rivera-Tinoco, R., Verbong, G., & Van der Zwaan, B. Historical variation in the capital costs of natural gas, carbon dioxide and hydrogen pipelines and implications for future infrastructure. *International Journal of Greenhouse Gas Control*, *5*(6), 1614-1623 (2011).

29. Wang, A., van der Leun, K., Peters, D., & Buseman, M. *European Hydrogen Backbone. How a dedicated hydrogen infrastructure can be created*. Technical Report Gas for Climate (2020).

30. Meylan, F. D., Moreau, V., & Erkman, S. CO2 utilization in the perspective of industrial ecology, an overview. *Journal of CO2 Utilization*, *12*, 101-108 (2015).

31. Morbee, J. International Transport of Captured CO_2: Who Can Gain and How Much?. *Environmental and Resource Economics*, *57*(3), 299-322 (2014).

32. Morbee, J., & Proost, S. Russian gas imports in Europe: how does Gazprom reliability change the game?. *The Energy Journal*, *31*, 4 (2010).

33. Morbee, J., Serpa, J., & Tzimas, E. Optimised deployment of a European CO2 transport network. *International Journal of Greenhouse Gas Control*, *7*, 48-61 (2012).

Supplementary Information 2 – The HyPE model

Principles and Methodology

Hydrogen Pathway Exploration model (HyPE) is a dynamic optimisation model that minimises the cost of global clean hydrogen supply and delivery, including technical, operational and resource-related constraints. HyPE aims at providing to the main energy system optimisation model a way to introduce potential hydrogen imports from neighbouring regions (notably North-Africa, Middle East and Ukraine). It provides cost-optimal production and trade routes for clean hydrogen, considering all potential production sites and possible transport options. The results consist in supply curves, indicating both the potential of hydrogen production per country and the associated costs following a levelised cost of hydrogen approach (LCOH) and a cost, insurance and freight logic (CIF – Supplementary Figure 8). The levelized cost of hydrogen adopts the life cycle costing methodology. It is defined as the sum of all the discounted fixed and variable costs necessary for the production of hydrogen over the expected lifetime of the installation, divided by the total volume produced during its lifetime. Cost, insurance and freight, as defined in Incoterms, means that the exporter delivers the product at the port of destination, so the cost at the loading port includes the cost of transport and logistics. The methodology builds on the full delivery value chain up to an entry point in Europe to determine the specific LCOH of each European importing route.

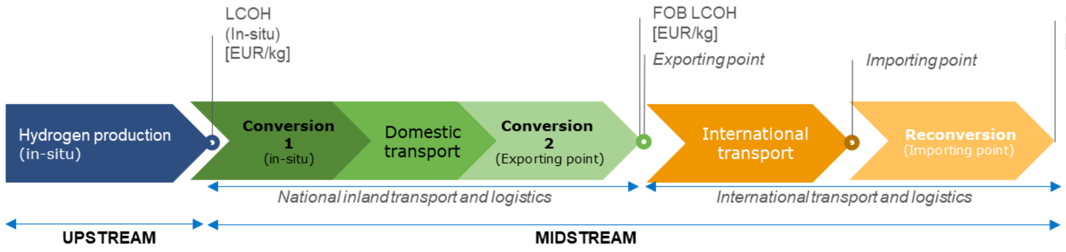

Supplementary Figure 8. Hydrogen trade value chain in the HyPE model. LCOH is the acronym for the levelized cost of hydrogen production, FOB (freight on board) represents the levelised cost of hydrogen at the export point, including the transport and conversion costs in the exporting region, and CIF (cost, insurance and freight) represents the landed cost of hydrogen in the importing region adding the cost of international transport and reconversion (if needed) on the FOB.

The two overarching principles of the HyPE model are:

1. Low-carbon European energy imports: decarbonisation of energy imports can be achieved via decarbonising imported natural gas (either pre-combustion or post-combustion), or by importing any other renewable or decarbonised gases (e.g., H_2/LH_2, P2G, Biomethane, etc.).

2. Technology neutrality of hydrogen production: Natural gas converted to hydrogen at import point/city gate or direct hydrogen imports[1].

The approach builds on linear programming to choose the most cost-efficient way to satisfy the hydrogen demand in the considered countries and provide the MIRET-EU model with least-cost renewable and low-carbon hydrogen potentials from each of the neighbouring countries, including potential demand hubs that might impact the availability of clean hydrogen for exports to Europe, notably China, Japan and Korea. This results in an optimisation model choosing the most cost-efficient way to supply hydrogen to Europe, considering different upstream options (e.g., renewable energy and natural gas), transport modalities (e.g., trailers,

pipeline and bunkers) and energy vectors (e.g., ammonia, liquified hydrogen and gasified hydrogen). The resulting cost structure is therefore driven by production costs, but also includes transport costs and conversion and reconversion costs depending on the transport technology and route. The cost-minimisation is performed in a country-neutral and technology neutral way.

Upstream activities: hydrogen production

Renewable hydrogen production from variable renewable energies

In HyPE, green hydrogen can be produced either via electrolysis of variable renewable energy sources (wind and solar power) or from processes based on biomass (biomass reformation, bio-pyrolysis) which can allow negative emissions. While hydrogen production from biomass can be economically less viable, it can provide negative emissions for offsetting residual CO_2 emissions and upstream and midstream methane emissions associated with natural gas-based low-carbon hydrogen.

The production of renewable hydrogen from wind and solar energy depends local factors such as the domestic wind and solar irradiation potentials, as well as on the availability of suitable land and water access. The methodology for the estimation of feasible solar and wind resources for the production of renewable hydrogen is based on Ruiz et al.[2] and Milbrandt and Mann[3].

To capture the available land for wind and solar resources a 2.5° decimal degree grid has been projected on the considered export regions (Supplementary Figure 9). For each cell both an annual wind speed hourly time series and an annual solar irradiation hourly time series[4,5] were determined at its centroid location based on 2016 data from the NASA MERRA-2 dataset. Using these timeseries, hourly wind and solar capacity factors are linked to hourly hydrogen from the weather data for the year 2016. For onshore wind turbines, a hub height of 130 meters and the Vestas V150 4000 turbine's power curve were considered to obtain the hourly wind capacity factors at every cell. Fixed ground-mounted PV systems with optimised tilt angles (as a function of the cell latitude) were considered to represent solar power plants in the model. The study considers the possibility to install off-grid dedicated single and/or hybrid systems. The hybrid system can possibly consist of three elements namely an electrolyser, a wind plant and/or a solar plant. The optimisation of the size of each component is handled within the model and based on techno-economic characteristics such as component costs and locational specific factors such as financing costs and natural resources. Consequently, depending on its location, the optimal configuration can either consist of only one power production source (solar PV or onshore wind) plus an electrolyser system or of a combination of both power sources plus an electrolyser system. For the optimally determined system configuration at every cell centroid, the corresponding levelized costs of renewable hydrogen are derived for each year.

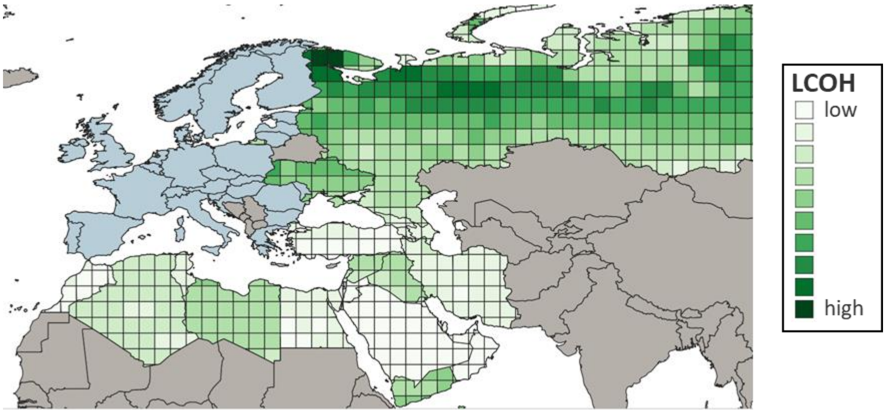

Supplementary Figure 9. Illustrative map of the considered cells around Europe and the calculated levelised cost of hydrogen (LCOH) in each cell. In this illustration, the lightest colours represent the least-cost hydrogen production cost, while the dark green tones represent the locations with the highest cost of hydrogen production. This figure is reproduced from the Hydrogen for Europe study's 2022 edition[6].

The maximum exploitable renewable potential builds the basis for the determination of the potential renewable hydrogen production volumes. Occupied land on every cell were analysed to obtain the available space for the installation of renewable energies and hence, to determine the potential production of hydrogen. Surfaces assigned to the categories of residential and industrial areas, as well as national parks and water bodies are considered to be non-usable for renewable energy production and hence were excluded from the technical potential calculations (Supplementary Figure 10). For the remaining areas, it is considered that only 5% of the land surface can be available for the deployment of solar PV. Power density describes the installable capacity over an area. We assume a 170W/m^2 of power density for PV modules and a 40% of farmland coverage by PV modules, leading to a 68W/m^2 of solar farm energy density[7]. For onshore wind, all available surfaces were assumed to be eligible for the deployment of onshore wind. A power density of 5 MW/km^2 was applied for this technology following Ruiz et al.[2]. To limit domestic transport costs and energy losses, cells within a maximum distance of 1000km to an international exit point (terminal or pipeline) were taken into account in the analysis as potential exporting locations given the absence of hydrogen domestic transport in the exporting countries considered.

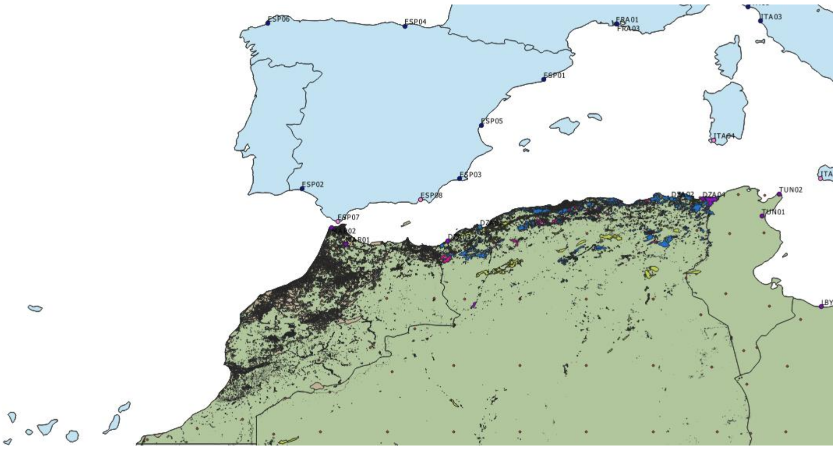

Supplementary Figure 10. Illustrative example on the determination of the maximum available space for the installation of renewable energies using land-use data. Black, blue, red and green dots represent the surface that cannot be used for wind and power installations. This figure is reproduced from the Hydrogen for Europe study's 2022 edition[6].

The maximum exploitable trajectory of national renewable energy that can be dedicated to hydrogen production follows maximum deployment rates constraints to mimic industrial and regulatory rigidities preventing the industry to be developed overnight. The obtained renewable potentials were verified against the international potential estimated by NREL[8].

The technology cost data for solar and wind power plants and the electrolysers for off-grid renewable hydrogen production are the same as the ones used in the MIRET-EU model in Supplementary Information 1. Using these data and the renewable potential data, the levelised cost of hydrogen production can be calculated following Equations 2 and 3.

$$\begin{cases} CAPEX_{tech,y}: & \text{Initial investments for a given production technology } tech \text{ on year } y \\ OPEX_{tech,y}: & \text{Maintenance and operational costs for a given } tech \text{ and year } y \\ WACC_{tech,y,country}: & \text{Weighted Average Cost of Capital in the } country \text{ and year } y \text{ per } tech \\ E_{tech,cell}: & \text{Annual energy output per } tech \text{ on a prodcution } cell \text{ in kilograms of hydrogen} \\ CF_{h,tech,cell}: & Capacity\ Factor - \text{ Energy produced out of one kW of capacity installed,} \\ & \text{in kWh, per hour } h, tech \text{ on a production } cell \\ \eta_{electrolysis}: & \text{Consumption of electricity of the electrolyser in kg/kWh} \\ lt_{tech}: & \text{Lifetime of the production technology } tech \text{ considered} \end{cases}$$

$$LCOH_{tech,y,country} = \frac{CAPEX_{tech,y} + \sum_{t=1}^{lt_{tech}} \frac{OPEX_{tech,y}}{\left(1+WACC_{tech,y,country}\right)^t}}{\sum_{t=1}^{lt_{tech}} \frac{E_{tech,cell}}{\left(1+WACC_{tech,y,country}\right)^t}} \tag{2}$$

$$E_{tech,cell} = \sum_{h=1}^{8760} CF_{h,tech,cell} \times \frac{1}{\eta_{electrolysis}} \tag{3}$$

Water availability and competition with other uses is a major topic in particular in regions with resource scarcity as in some parts of Middle East, North Africa, Sub-Saharan Africa, Australia and Chile. Concerning the current study, Middle East and North Africa are the key clean hydrogen exporters to Europe. To internalise this issue, we follow a similar approach as that commented by IRENA in its global hydrogen trade outlook[9]. We assume that for acceptability reasons all water consumption of electrolysers comes from seawater desalination. Accordingly, only sites within 300 km from the sea are considered and the associated costs of water supply are included in the LCOH calculations.

Water desalination is already supplying about 95 million m^3/day of water and producing 142 million m^3/day of brine[10]. Curto et al.[11] discuss the state of play of desalination technologies and affirms the next frontier for key commercial technologies such as reverse osmosis (RO), multi-stages flash desalination (MSF) and multi-effect distillation (MED), is to be powered by renewable sources. We base our water cost calculations on the technoeconomic figures reported by them for the different technologies considered in the model.

We estimate water cost by adopting an amortisation logic to desalination plants which includes capital and operational expenditures over its economic lifetime. We assume that the electricity used by desalination units comes from the power grid of the countries considered. Hence, our operational costs include costs of the electricity used, and the expenditures for offsetting the

associated emissions of grid electricity. With the data of the electricity mix in each of the countries we estimate average electricity prices. The carbon intensity of the electricity supply leads to a carbon footprint of the desalinated water. We apply a carbon tax in line with the EU ETS, reaching €250/tCO_2 by 2050 penalising the associated emissions, and more importantly, to implement a level-playing field between countries with different electricity mixes (this is currently being discussed in the design of carbon border adjustment mechanisms). These values are compared to the water production cost estimated by the World Bank[12] and calibrated based on these values.

Renewable hydrogen production from biomass

The potential renewable hydrogen production from biomass highly depends on the availability of natural feedstock resources. Only Russia and the Ukraine have substantial biomass potential in the considered geographical scope of exporting countries. As Russia has been excluded from being a trading partner of Europe, only Ukraine was considered for the production of hydrogen from biomass.

Another important aspect for hydrogen from biomass is the ability to transport the feedstock to the production facility which is considered to be centralised and located in the proximity of an exporting point. Therefore, similar to the production of hydrogen from wind and solar, a 2.5 decimal degree grid was projected on the lands to capture the impact of transportation costs and availability on the production potential of hydrogen from biomass.

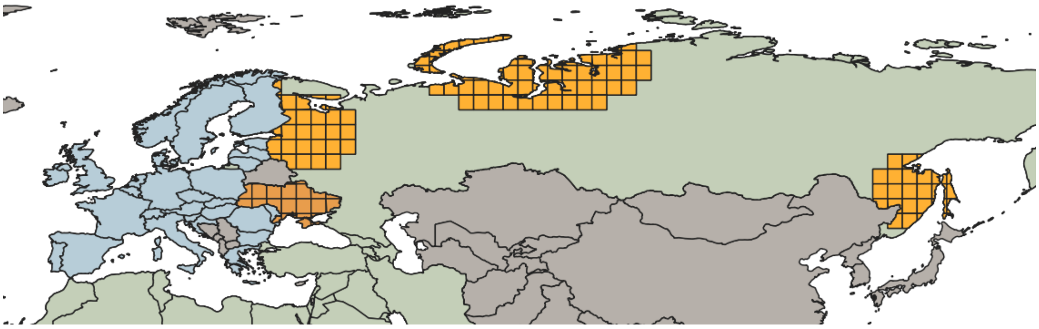

Supplementary Figure 11. Potential biomass-based renewable hydrogen exporters to Europe in the HyPE model. Current study only considers Ukrainian biomass-based renewable hydrogen as potential hydrogen imports to Europe. This figure is reproduced from the Hydrogen for Europe study's 2022 edition[6].

The total biomass feedstock availability is considered to be uniformly distributed on the regions considered. Furthermore, only solid forest residues are considered as feedstock for the production of hydrogen as it is the only easily transportable biomass with an economically viable energy density. Considering its volume and energy density, the maximum freight range admissible for biomass that is considered in this study is 700 km. The resulting volumes of biomass that are available for the hydrogen production in the resulting regions are given in Supplementary Table 6. The maximum distances of 700 km allow to compare the transportation of biomass with the transportation of coal so that associated freight cost is assumed to be around €0.425/kgH_2/1000km[13]. Moreover, only 50% of the overall available biomass is assumed to be

available for the production of hydrogen for exports to account for other domestic uses in competition.

Supplementary Table 6. Available biomass stocks considered in HyPE that can be potentially used to produce renewable hydrogen for exports to Europe[14].

Area	Available biomass feedstock (TWh)
**Ukraine**	29.9

The production of hydrogen from biomass is assumed to take place in a centralised gasification plant close by an exporting point. The resulting levelized costs of hydrogen consists accordingly of the costs related to the production facility, the transportation of biomass as well as feedstock costs. Bio-feedstock cost is considered to be $6.5/GJ[15]. The gasification facilities are assumed to operate at a load factor of 85% throughout their technical lifetime (30 years).

Low-carbon hydrogen production from natural gas

Only imports of low carbon hydrogen from countries that produce natural gas domestically were considered (i.e., Algeria, Azerbaijan, Iran, Qatar, Russia, Egypt, United Arab Emirates and Saudi Arabia). Given that natural gas infrastructure is well developed in the countries considered, production facilities are assumed to be installed near the location of the current exit points for natural gas trade (pipeline and/or terminal), so to avoid additional inland transport costs.

Two set of technologies to produce low-carbon hydrogen were assessed:

- Reformers with CCS: steam methane reforming (SMR), autothermal reforming (ATR) and gas heated reforming (GHR), all with carbon capture and storage (CCS) were considered. Full cost was considered assuming rock formations where available within a reasonable distance around the production sites[16] to estimate an average cost related to CO_2 transport and storage. It was assumed that CO_2 storage capacities at those sites were at least 10 Mt per year which would lead to a transport and storage cost of around €11.4/tCO_2 (after considering economies of scale) based on the H21 North of England report[17].
- Methane pyrolysis (carbon black co-product revenues included): Same as for the European hydrogen production alternatives, methane pyrolysis was assumed to be commercially available from 2030 onwards.

Scenario analysis on natural gas production, domestic consumption and trade were conducted in each of the gas-rich MENA countries to assess the possible availability of natural gas for producing hydrogen. Additionally, Azerbaijan (AZE) is expected to produce around 50 bcm of natural gas in 2025 at the completion of the SCP/TANAP/TAP project, where we expect around 20% this production could be used to produce low carbon hydrogen to the EU market by 2025.

Given the existing infrastructure development programmes, no further projections regarding the evolution of the exporting shares are considered.

Wellhead cost of natural gas production was assumed to be the average breakeven price in each exporting country. Breakeven gas prices were estimated by calculating the percentage difference with respect to country average breakeven oil prices from the International Monetary Fund and applying it to gas producing countries assuming an average of $2.4/MMBtu as the basis. This value corresponds to the average US dry gas wellheads breakeven values reported by BNEF[18]. For Algeria, with a declining export trend[19], the breakeven cost assumed was that of a dry gas Africa shore well. Tests have been conducted to provide robustness to this assumption and we have obtained that LOCH are within the +/-10% range for all countries with average breakeven prices changing in the $2/MMBtu to $2.9/MMBtu window. The obtained range of breakeven gas prices were verified by benchmarking them against typical average wellhead cost of basins of similar type for each region (i.e., onshore, deep, shallow, ultra-deep). They don't include any national tax scheme.

Price of carbon black (by-product of methane pyrolysis) was assumed constant at €100/tC, and compensation for unabated CO_2 emissions, as well as upstream methane emissions, for both reformers with CCS (95% of assumed capture rate) and methane pyrolysis were accounted for by assuming economic offsets given by a CO_2 emission cost trajectory proposed by the IEA for developing economies. All the technology cost data related to reformers and pyrolysis plants are the same as in the Supplementary Information 1.

Midstream: hydrogen transport

The competitiveness of hydrogen imports depends on the availability of the transport infrastructure. Depending on the distance between production and delivery points, several transportation paths are currently envisaged and integrated into the modelling framework in accordance with the overall technology-neutral approach. For national inland transport of hydrogen, trucks, either with compressed hydrogen or ammonia are considered. For international transport, hydrogen pipelines, ammonia shipping and liquified hydrogen (LH_2) have been considered (Supplementary Figure 12).

International hydrogen transport via ships

Liquified hydrogen terminals have been added to the model as possible entry points in Europe for hydrogen. As the natural gas consumption in Europe drops, EU LNG terminals are assumed to see their utilisation rates dropping from 2025 onwards. Nevertheless, current energy crisis underpinned by the Russian invasion of Ukraine led to increased LNG consumption in Europe in the last year, and this increase is set to continue in 2023[20]. However, we assume that this situation will not persist after the end of current decade[6]. We therefore make the assumption that the refurbishment of LNG terminals to handle liquified hydrogen (LH_2) is an option from 2030 onwards. The older terminals in countries with the lowest utilisation rates have been assumed to be the first candidates for repurposing to hydrogen. The availability timeline is then made by a combination of the commissioning date and the forecasted utilisation rate of the terminals (Supplementary Table 7). Refurbishment of existing terminals is preferred to build new ones as it allows to save around 25% of investment costs from new builds[21]. In terms of volumetric capacity, liquified hydrogen terminals are supposed to handle each year the same

volume of liquid gas than the previous LNG terminals did. On an energetic point-of-view, it means only 40% of the energy capacity of the LNG terminal is available when refurbished to hydrogen.

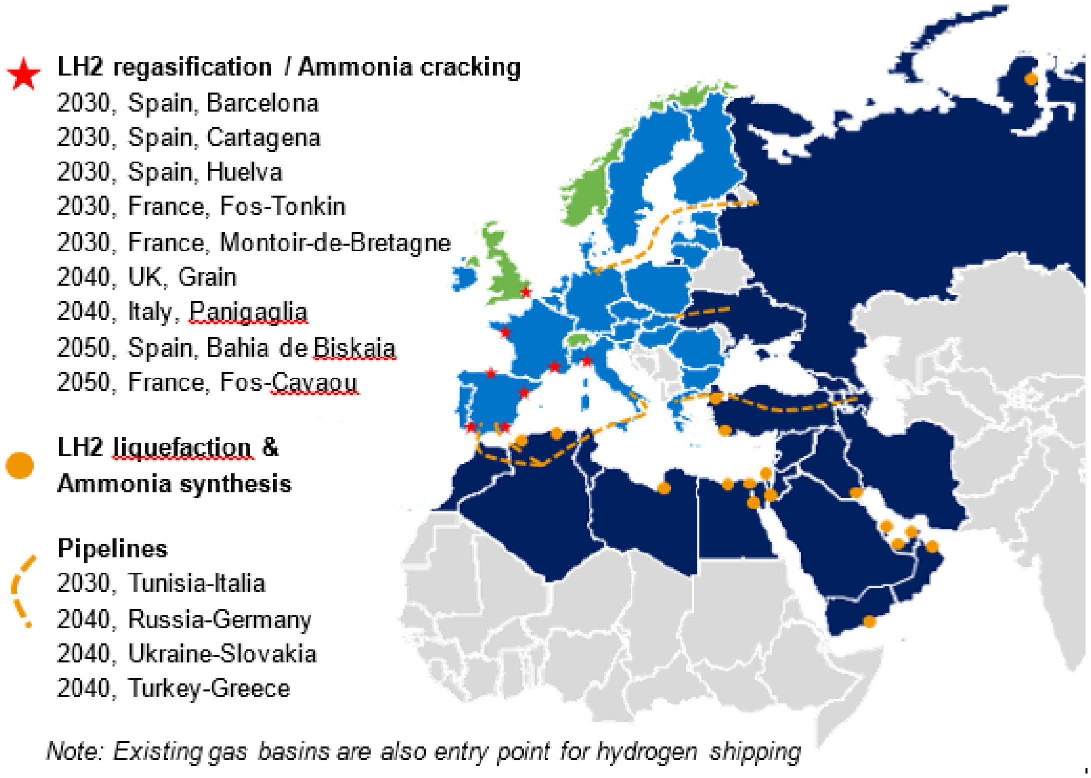

Supplementary Figure 12. Geographical scope considered for hydrogen imports to Europe and alternative routes based on Seck et al.[22] and Hydrogen for Europe[6].

Supplementary Table 7. Considered liquid hydrogen and ammonia import terminals' availabilities in Europe, including the refurbishment year assumptions, capacity and age of each terminal.

Country	Terminal	Capacity (TWh)	Start year	Assumed refurbishment year	Terminal age (in 2020)
**Spain**	Barcelona	180	1969	2030	51
**Italy**	Panigaglia	37	1971	2045	59
**France**	Fos Tonkin	31	1972	2030	48
**France**	Montoir-de-Bretagne	105	1980	2030	40
**Spain**	Huelva	124	1988	2030	32
**Spain**	Cartagena	124	1989	2030	31
**Spain**	Bahia de Biskaia	120	2003	2050	37
**UK**	Grain	214	2005	2040	15
**France**	Fos Cavaou	86	2010	2050	30

Capacity of liquified ammonia terminals might as well be expanded, justifying the choice to consider this carrier as a feasible hydrogen importing option in Europe. Ammonia (NH_3) is seen as an effective hydrogen carrier for long distance shipping. It presents the advantage to be liquid at higher temperature than LNG or liquified hydrogen, and its higher energy density allows it to compete in terms of costs against liquified hydrogen. From a volumetric point-of-view,

ammonia presents an energy density 1.7 times higher than liquified hydrogen[23]. This means that for the volume transported per year, ammonia regasification plants transport 70% more energy than a liquified hydrogen plants with equivalent capacity. Based on these elements, a timeline has been created to model the evolution of energy-related ammonia importing terminals in Europe. Similar to shipping liquified hydrogen, ammonia is assumed to be reconverted to hydrogen at the importing port. Thus, a final step of catalytic cracking of ammonia is considered in the LCOH estimate for this route. The international trade of ammonia is well established but despite promising prospects for ammonia as a shipping fuel, such a use is not expected before 2035[24]. Therefore, considering ships fuelled and transporting ammonia (decarbonised) is essential to be consistent with the hypothesis of low-carbon EU energy imports.

International hydrogen transport via pipelines

Regarding the cross-border gas interconnectors, the assumptions adopted are based on the European Hydrogen Backbone study[25].based on this study, we assume that a dedicated hydrogen network in the EU is progressively built by repurposing some natural gas pipelines and building new ones. Deployment of such network would start from key industrial clusters in 2030, expand to EU interconnectors by 2035 and reach some non-EU interconnectors by 2040. For calculating an LCOH component of hydrogen transmission by pipeline, assumptions on which and by when each interconnector is available, its route, length and capacity are key. The retrofitted pipeline capacity assessed in the European Hydrogen Backbone study and its timeline have been considered as inputs of the model (Supplementary Table 8). More specifically six pipelines have been considered for hydrogen imports, allowing both low-carbon and renewable hydrogen to be imported into Europe. Repurposed pipelines are supposed to handle each year almost the same energy capacity as the previous gas pipelines did (assuming a Wobbe index between 41 and 47 MJ/Nm^3 for natural and 40.65 MJ/Nm^3 for gaseous hydrogen[26]). Only one injection point has been considered for each country. It is supposed to be located according to the gas network topology and existing compression stations (for Azerbaijan and Algeria, additional injection points have been added where pipelines start).

Supplementary Table 8. Considered retrofitted natural gas pipelines for hydrogen transport. Following European REPowerEU plan in response to Russian invasion of Ukraine, the pipelines between Russia and Europe have been excluded from the trade options in this study.

Entry point (ENTSOG)	Type	Code Country (entry point)	Code Country (exit point)	Start year	Infrastructure name	Max volume (MTPA H2)	Length (km)
207	Pipeline	ESP	MAR	2040	MEG	4.8	45
207	Pipeline	ESP	DZA	2040	MEG	4.8	1082
208	Pipeline	ESP	DZA	2040	Medgas	3.1	210
208	Pipeline	ESP	DZA	2040	Medgas	3.1	757
209	Pipeline	ITA	TUN	2030	Transmed	6.2	155
209	Pipeline	ITA	DZA	2030	Transmed	6.2	1075
222	Pipeline	GRE	TUR	2040	TANAP	3.1	110
222	Pipeline	GRE	AZE	2040	TANAP	3.1	2496
218	Pipeline	SVK	UKR	2040	Kyev - Western Border Pipeline	9.9	650

The cost assumptions of hydrogen transport are presented in Supplementary Tables 9 and 10.

Supplementary Table 9. The cost assumptions of hydrogen transport via ships, including compression, inland transport via trucks, liquefaction and/or ammonia synthesis, exporting terminal costs, international shipping cost, importing terminal cost and regasification and/or ammonia cracking costs (own calculations based on data from IEA[27], DOE[28], Fúnez Guerra et al.[29], Ikäheimo et al.[30] and Davenne et al.[31]).

	**Production**	**Conversion 1 (production point)**	**Domestic transport**	**Conversion 2 (exporting point)**		**International transport**		**Reconversion (Importing point)**
**A.** (from 2020)	**From all Sources available in the cell** Depends on the technology and resources available	**Compression**	**Gasified trucks**	**Liquefaction**	New dedicated exporting terminals including storage	**Liquified hydrogen shipping**	Refurbished dedicated importing terminals	**Hydrogen regasification**
LCOH Contribution (EUR17/Kg)		A function of distance: LCOH = 2.65 D + 0.27 D: Distance (1000 Km) ; Max. range: 300 Km		2.03		A function of distance: LCOH = 0.10 D + 0.84		1.30
**B.** (from 2030)		**Compression**	**Gasified trucks**	**Ammonia synthesis & liquefaction**		**Liquified Ammonia shipping**		**Ammonia catalytic cracking**
LCOH Contribution (EUR17/Kg)		A function of distance: LCOH = 2.65 D + 0.27 D: Distance (1000 Km) ; Max. range: 300 Km		1.53		A function of distance: LCOH = 0.09 D + 0.17		1.80
**C.** (from 2030)		**Ammonia synthesis**	**Liquid Ammonia trucks**	**N/A**		**Liquified Ammonia shipping**		**Ammonia catalytic cracking**
LCOH Contribution (EUR17/Kg)		1.53	A function of distance: LCOH = 0.66 D + 0.04 D: Distance (1000 Km) Max. range: 1000 Km	-		A function of distance: LCOH = 0.09 D + 0.17		1.80

Supplementary Table 10. Cost assumptions for hydrogen transport via pipelines, including compression, transport via trucks and pipeline costs (own calculations based on data from IEA[27], DOE[28], Fúnez Guerra et al.[29], Ikäheimo et al.[30] and Davenne et al.[31]).

	**Production**	**Conversion 1 (production point)**	**Domestic transport**	**Conversion 2 (exporting point)**		**International transport**
**D.** **(from 2040)**	**From all Sources**	**Compression**	**Gasified trucks**	**Compression**	**Injection point**	**Hydrogen pipelines**

LCOH Contribution (EUR17/Kg)	**available in the cell**	A function of distance:<br>*LCOH = 2.61 D + 0.25*<br>*D: Distance (1000 Km) ; Max. range: 300 Km*				A function of distance:<br><br>LCOH = 0.55 D + 0.06<br>D: Distance (1000 Km)
**E.**<br>(from 2040)	Depends on the technology and resources available	**Ammonia synthesis**	**Liquid Ammonia trucks**	**Ammonia catalytic cracking**		
LCOH<br>Contribution<br>(EUR17/Kg)		*1.53*	A function of distance:<br>*LCOH = 0.65 D + 0.04*<br>*D: Distance (1000 Km) ; Max. range: 1000 Km*	*1.80*		
**F.**<br>**(from 2050)**		**Compression**	**Gasified trucks**	**Compression**		
LCOH<br>Contribution<br>(EUR17/Kg)		A function of distance:<br>*LCOH = 2.52 D + 0.23*<br>*D: Distance (1000 Km) ; Max. range: 300 Km*				
**G.**<br>(from 2050)		**Ammonia synthesis**	**Liquid Ammonia trucks**	**Ammonia catalytic cracking**		
LCOH		*1.53*	A function of distance:<br>*LCOH = 0.64 D + 0.04*<br>*D: Distance (1000 Km) ; Max. range: 1000 Km*	*1.80*		

Country-specific WACC

As any investment, the deployment of hydrogen facilities has inherent risk that directly translate into cost of capital. Additionally, undertaking such investments in countries with somehow challenging local regulation needs to be factored into the LCOH calculation. We therefore consider country specific risk premiums, estimated by the relative ratio of the Ease of Doing Business scores[32] of each country against the EU average. Future values were linearly extrapolated according to the historic trend and corrected by a deflator. This methodology allows to approximate a country-dependent risk adjusted weighted average cost of capital (WACC) for the LCOH calculation.

Compared to the average EU-27 WACC of 8%, we consider a range going from 6% in 2018, in economically stable countries such as the United Arab Emirates, to more than 15% in countries such as Yemen or Libya, that face long-lasting instability (Supplementary Figure 13**Error! Reference source not found.**). As an indicative figure, when varying country-specific WACC by +/-20% it results on a difference on LCOH of within the 5% range for low-carbon hydrogen technologies (technologies with higher shares of CAPEX on their LCOH would be more sensitive to variations of the WACC).

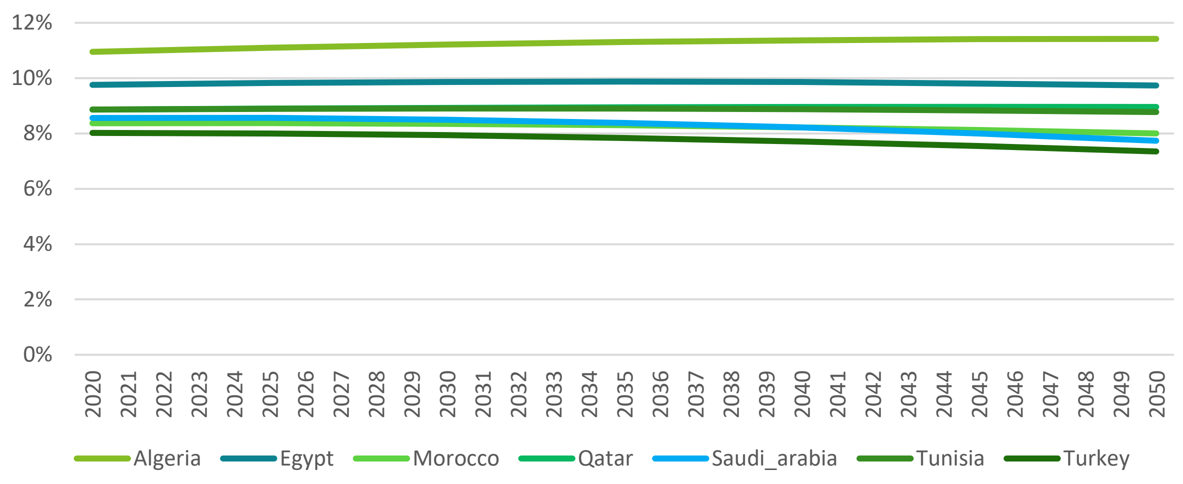

Supplementary Figure 13. Country-specific WACC (weighted average cost of capital) used in LCOH calculations. These values are used based on the World Bank's Ease of Business scores[32] and their extrapolation through the period to 2050.

Supplementary References

1. ENTSO-E & ENTSO-G. *TYNDP 2020: Joint Scenario Report* (2020).

2. Ruiz, P., Nijs, W., Tarvydas, D., Sgobbi, A., Zucker, A., Pilli, R., ... & Thrän, D. ENSPRESO-an open, EU-28 wide, transparent and coherent database of wind, solar and biomass energy potentials. *Energy Strategy Reviews*, *26*, 100379 (2019).

3. Milbrandt, A., & Mann, M. *Potential for Producing Hydrogen from Key Renewable Resources in the United States* (No. NREL/TP-640-41134). National Renewable Energy Lab.(NREL), Golden, CO, United States (2006).

4. Pfenninger, S., & Staffell, I. Long-term patterns of European PV output using 30 years of validated hourly reanalysis and satellite data. *Energy*, *114*, 1251-1265 (2016).

5. Staffell, I., & Pfenninger, S. Using bias-corrected reanalysis to simulate current and future wind power output. *Energy*, *114*, 1224-1239 (2016).

6. Hydrogen 4EU. *Charting Pathways to enable net zero* – 2022 edition (2022). https://www.hydrogen4eu.com/.

7. Tröndle, T. Supply-side options to reduce land requirements of fully renewable electricity in Europe. *PloS one*, *15*(8), e0236958 (2020).

8. Connelly, E., Penev, M., Elgowainy, A., & Hunter, C. Current Status of Hydrogen Liquefaction Costs. *DOE Hydrogen and Fuel Cells Program Record*, 1-10 (2019). https://www.hydrogen.energy.gov/pdfs/19001_hydrogen_liquefaction_costs.pdf.

9. IRENA. *Global Hydrogen Trade to Meet the 1.50C Climate Goal. Part 1: Trade Outlook for 2050 and Way Forward*. International Renewable Energy Agency, Abu Dhabi, UAE (2022). https://www.irena.org/publications/2022/Jul/Global-Hydrogen-Trade-Outlook.

10. Jones, E., Qadir, M., van Vliet, M. T., Smakhtin, V., & Kang, S. M. The state of desalination and brine production: A global outlook. *Science of the Total Environment*, *657*, 1343-1356 (2019).

11. Curto, D., Franzitta, V., & Guercio, A. A review of the water desalination technologies. *Applied Sciences*, *11*(2), 670 (2021).

12. World Bank *The role of Desalination in an Increasingly Water-Scarce World*. World Bank. Washinhgton, DC United States (2019). https://openknowledge.worldbank.org/server/api/core/bitstreams/79d2f14b-3e5b-539f-bec5-e91a8574c238/content

13. Argus *Russian Coal 2017* (2017). https://www.argusmedia.com/ja/news/1791377-argus-russian-coal--russia-to-boost-its-share-in-global-coal-trade?page=1.

14. IRENA *Renewable energy prospects for Ukraine. International Renewable Energy Agency*. Abu Dhabi, UAE (2015). https://www.irena.org/publications/2015/Apr/Renewable-Energy-Prospects-for-Ukraine.

15. IRENA. *REmap 2030 Renewable Energy Prospects for Russian Federation*. Abu Dhabi, UEA (2017). *http://www.irena.org/remap*.

16. IPCC, E. *Carbon Dioxide Capture and Storage*. IPCC Special Report (2005).

17. H21. *H21 North of England*. H21. Leeds, The United Kingdom (2018). https://h21.green/projects/h21-north-of-england/.

18. Bloomberg New Energy Finance. *Sustainable Energy in America: Factbook* (2019). https://bcse.org/wp-content/uploads/2019-Sustainable-Energy-in-America-Factbook.pdf.

19. Aissaoui, A. *Algerian Gas: Troubling Trends, Troubled Policies* (2016).

20. IEA (2023). Natural gas supply and demand balance… https://www.iea.org/reports/background-note-on-the-natural-gas-supply-demand-balance-of-the-european-union-in-2023

21. Songhurst, B. LNG plant cost escalation (2014). https://doi.org/10.26889/9781907555947.

22. Seck, G. S., Hache, E., Sabathier, J., Guedes, F., Reigstad, G. A., Straus, J., Wolfgang, O., Ouassou, J.A., Askeland, M., Hjorth, I., Skjelbred, H.I., Andersson, L.E., Douguet, S., Villavicencio, M., Trüby, J., Brauer, J. & Cabot, C. Hydrogen and the decarbonization of the energy system in Europe in 2050: A detailed model-based analysis. *Renewable and Sustainable Energy Reviews*, *167*, 112779 (2022).

23. Aziz, M., Wijayanta, A. T., & Nandiyanto, A. B. D. Ammonia as effective hydrogen storage: A review on production, storage and utilization. *Energies*, *13*(12), 3062 (2020).

24. Brinks, H., & Hektor, E. A. Ammonia as a marine fuel. In *Proceedings of the Alternative Fuels Online Conference, Online*, Vol. 15 (2020).

25. Wang, A., van der Leun, K., Peters, D., & Buseman, M. European hydrogen backbone: How a dedicated hydrogen infrastructure can be created (2020).

26. Haeseldonckx, D., & D'haeseleer, W. The use of the natural-gas pipeline infrastructure for hydrogen transport in a changing market structure. *International Journal of Hydrogen Energy*, *32*(10-11), 1381-1386. https://doi.org/10.1016/j.ijhydene.2006.10.018 (2007).

27. IEA. The Future of Hydrogen (2019). https://www.iea.org/fuels-and-technologies/hydrogen.

28. Connelly, E., Penev, M., Elgowainy, A., & Hunter, C. Current Status of Hydrogen Liquefaction Costs. *DOE Hydrogen and Fuel Cells Program Record*, 1-10. https://www.hydrogen.energy.gov/pdfs/19001_hydrogen_liquefaction_costs.pdf (2019).

29. Guerra, C. F., Reyes-Bozo, L., Vyhmeister, E., Caparrós, M. J., Salazar, J. L., & Clemente-Jul, C. Technical-economic analysis for a green ammonia production plant in Chile and its subsequent transport to Japan. *Renewable Energy*, *157*, 404-414 (2020).

30. Ikäheimo, J., Kiviluoma, J., Weiss, R., & Holttinen, H. Power-to-ammonia in future North European 100% renewable power and heat system. *International Journal of Hydrogen Energy*, *43*(36), 17295-17308. https://doi.org/10.1016/j.ijhydene.2018.06.121 (2018).

31. Jackson, C., Fothergill, K., Gray, P., Haroon, F., Makhloufi, C., Kezibri, N., Davey, A., Hote, O.L., Zarea, M. and Davenne, T. Ammonia to green hydrogen project. *Feasibility study*, *1* (2020).

32. Doing Business. *Comparing Business Regulation in 190 Economies*. International Bank for Reconstruction and Development. The World Bank, Washington (2020). http://www.doingbusiness.org/ExploreTopics/PayingTaxes/CompareAll.aspx

Supplementary Information 3 – Methane emissions across the natural gas value chain

Principles and Methodology

This paper describes the methodological framework for estimating the methane (CH_4) emission factors (EF) for gas production and consumption. In total over 150 EF have been estimated covering the following:

a) all the countries mentioned in Supplementary Table 11.
b) different parts of the natural gas value chain
    - Upstream – Exploration, production, gas gathering and boosting, gas processing
    - Downstream – Gas transmission and gas distribution
    - LNG – Liquefaction, LNG carrier (transport) and LNG regasification
c) The period between 2019 and 2050: 2019, 2030, 2035, 2040, 2045 and 2050
d) Three different scenarios

The methane emission scenarios considered:

i. Current emissions (CEF): EF is estimated using the current best understanding of methane emission from the oil and gas sector. The estimated EF is considered flat from the year of analysis until 2050. These EF are used as the preliminary year EF (2019) for the two other cases.
ii. Harmonized pledges (HP): International pledges and country level targets and policies for methane and greenhouse gas reduction are considered to estimate the impacts on EF from the preliminary year (2019) to 2050.
iii. Best available technologies (BAT): This case represents the EF on applying the best available technologies for methane abatement. Industry targets set in the countries assessed, or global industry targets for BAT are considered to estimate the impacts on EF from the preliminary year (2019) to 2050.

Supplementary Table 11. Countries for which EF were assessed

Country category	Countries
Countries exporting natural gas (via pipeline) or exporting low carbon hydrogen to Europe	Algeria, Azerbaijan, Bahrain, Egypt, Iran, Iraq, Kuwait, Libya, Oman, Qatar, Russia, Saudi Arabia, United Arab Emirates (UAE)
Countries exporting LNG to Europe	Algeria, Angola, Australia, Egypt, Nigeria, Peru, Qatar, Russia, Trinidad and Tobago, United States of America (USA)
European producing countries	Denmark, Germany, Italy, Netherlands, Poland, Romania, UK, Norway
Importing countries	Austria, Belgium, Czech, Denmark, France, Finland, Germany, Greece, Italy, Ireland, Netherlands, Poland, Portugal, Romania, Slovakia, Slovenia, Spain, UK, Switzerland, Bulgaria, Croatia, Cyprus, Estonia, Latvia, Lithuania, Malta, Luxembourg, Hungary, Sweden

Selection of data sources for calculation of current methane emissions

While estimating the EF for the gas value chain, the first step is to understand the boundaries associated with the methane emissions (Supplementary Figure 14).

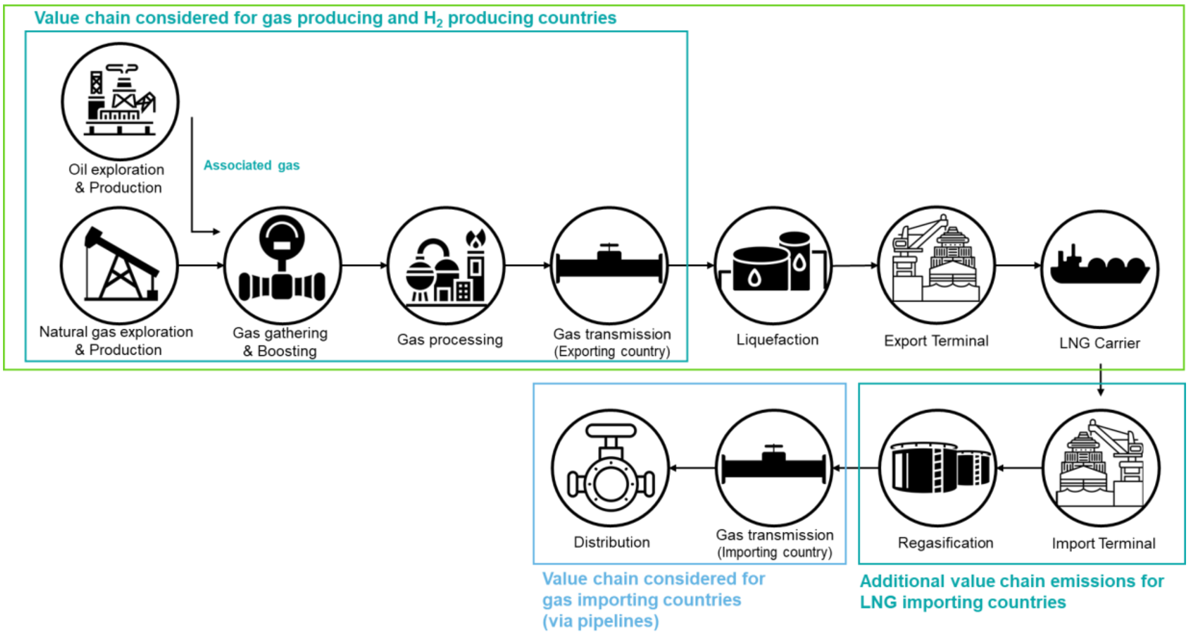

Supplementary Figure 14. Gas value chain steps considered for calculating the emission factors. The figure is based on Hydrogen for Europe study[1].

Country specific academic papers[2,3], national inventories[4] and IEA[5] data have been assessed to estimate the current emissions from the oil and gas sector for each country. While some countries have several academic papers and research work done on estimating the CH_4 emissions, some countries have very limited data available on the CH_4 emissions (Supplementary Table 12). A decision tree and other important considerations were used to select the best available source for each country (Supplementary Figure 15).

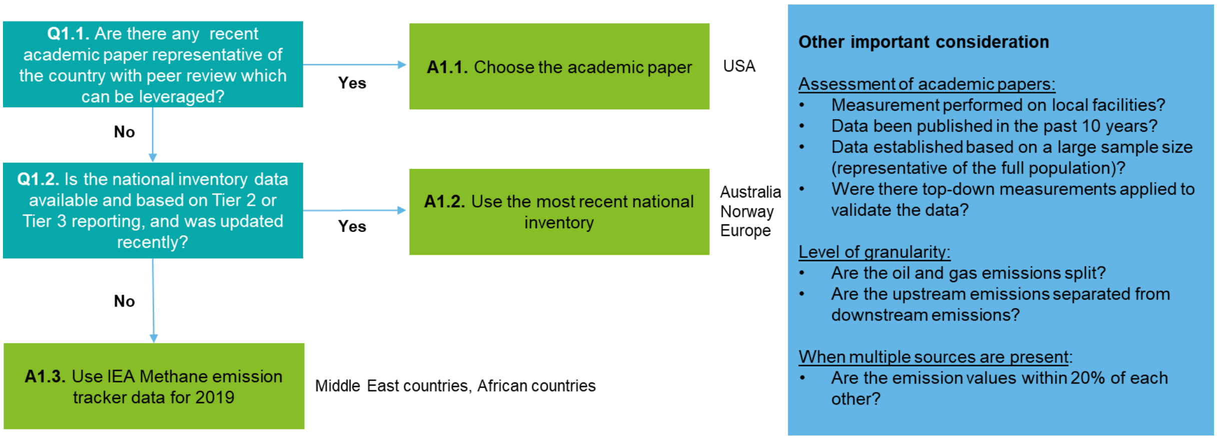

Supplementary Figure 15. Decision tree to identify the best source for the methane emissions

Supplementary Table 12. The final list of sources used for the estimation of current emissions for each country

Country	*Chosen source for emission*
Algeria, Angola, Egypt, Nigeria, Peru, Qatar, Russia	IEA (2019)[5] – for upstream oil and gas and downstream emissions<br>Carbon Limits expertise – for LNG Carrier EF<br>EPA Facility level data[6], Atlantic LNG facility[7], Melkoya facility[8] – for LNG Liquefaction emissions
Azerbaijan, Bahrain, Iran, Iraq, Kuwait, Lybia, Oman, Saudi Arabia, UAE	IEA (2019)[5] – for upstream oil and gas and downstream emissions

Australia	UNFCCC (2019)[9] – for upstream oil and gas and downstream emissions<br>Carbon Limits expertise – for LNG Carrier EF<br>EPA Facilities[10], Atlantic LNG facility[7], Melkoya facility[8] – for LNG Liquefaction
Norway	Carbon Limits expertise – for LNG Carrier emissions<br>Norsk Olje og Gass (2021)[10] – for upstream oil and gas emissions<br>Melkoya facility[8] – for LNG Liquefaction emissions
Trinidad and Tobago	IEA (2019)[5] – for upstream oil and gas and downstream emissions<br>Carbon Limits expertise – for LNG Carrier EF<br>Atlantic LNG facility[4]– for LNG Liquefaction emissions
USA	Alvarez et al. (2015)[2], Zhang et al. (2020)[11] – for upstream oil and gas and downstream emissions<br>Carbon Limits expertise – for LNG Carrier emissions<br>EPA Facilities[5] – for LNG Liquefaction emissions
Europe excluding Denmark, Germany, Italy, Netherlands, Norway, Poland, Romania, and UK (assessed separately, as gas producing countries in Europe)	UNFCCC (2019)[9] – for transmission and distribution emissions<br>Marcogaz (2018)[10] – for LNG unloading and regasification EF
Denmark, Italy, UK, Netherlands, Poland, Romania	UNFCCC (2019)[13] – for upstream and downstream (transmission and distribution) oil and gas emissions.<br>For associated gas - Danish Energy Agency[14] (Denmark),<br>Ministero della transizione ecologica[13] (Italy), Digest of UK Energy statistics (DUKES)[15] (UK)

For countries with the same source of information for emissions, a similar methodology was followed to estimate the EF along the gas value chain. It is to be noted that the HP and the BAT cases use the 2019 current emission factors as the preliminary year value. The following sections explain the calculations associated with the EF estimation.

Calculating upstream EF of exporting countries in CEF scenario. For countries where IEA[4], national level databases are the chosen source of information, the estimation steps used to assess the total upstream EF have been presented below.

Step A.1 – Estimating upstream non-associated gas emissions. This step only concerns non-associated gas, that is natural gas produced in dedicated gas wells. The total methane emissions are the sum of all the methane emissions categorized under natural gas production in IEA[4]: onshore and offshore fugitive, venting and flaring emissions. Upstream non-associated gas emissions (A) can be calculated using the Equation (4).

$$A = \Sigma\ (Fugitive, Venting, Flaring\ CH_4\ emissions)_{onshore} + \Sigma\ \ (Fugitive, Venting, Flaring\ CH_4\ emissions)_{offshore} \quad (4)$$

Step A.2 – Estimating associated gas (APG) emissions. To estimate the emissions related to the production of associated gas joining the natural gas value chain (marketable APG) from the oil production, some methane emissions from oil production must be attributed to marketable APG. This is done using the energy ratio of APG and oil produced in the country. The data for estimating the volume of APG produced per country is not easily available. Hence the regional APG production volumes have been leveraged, using IEA data[16]. Using this data, the ratio of regional APG produced to the total gas produced in the region is estimated using the Equation (5). The regional level data were swapped for country level data, where available.

$$Regional\ APG\ to\ gas\ ratio = \frac{Regional\ associated\ gas\ marketable}{Regional\ associated\ gas\ marketable + non-associated\ gas\ produced\ in\ region} \quad (5)$$

The regional APG to gas ratio is multiplied by the energy ratio of gas to oil produced in the country, to determine the part of the oil emissions that must be attributed to associated gas marketed. Lastly, the associated and non-associated gas emissions are summed and divided by total volume of gas produced (associated and non-associated) to obtain the EF.

For the case of USA, where academic papers are available with country-specific emissions data, the study Alvarez et al.[1] has been chosen as the main source of information for emissions in the US natural gas value chain. This study is the most recent in assessing the full country emissions and based on a significant number of measurements. It uses ground-based, facility-scale measurements and aircraft observation methods for estimating the emissions. One of the main areas with high methane emissions in the US is the Permian basin. Few academic research papers have been published since 2018, that assess methane emissions from this basin. The study by Zhang et al.[11] has been used as the main source of information for methane emissions in the Permian basin. To have an accurate representation of emissions from the entire country, the values from Alvarez et al.[1] have been adjusted, based on the Zhang et al.[11].

Alvarez et al.[1] study is used as the basis to estimate the oil and gas emissions in 2015. Emissions from the largest basin (the Permian basin) have been removed from the estimate proportionally to the Permian production in 2015. The remaining emissions have been projected from 2015 to 2018 (separately for oil and gas) using the evolution of methane emissions between 2015 and 2018. A change in methane emissions from 2015 to 2018 of -5.51% for gas and -1.34% for oil were estimated. Using these emissions, the steps mentioned in the sub-section above were applied to estimate the upstream EF for the US.

Calculating downstream EF of exporting countries in CEF scenario. For countries where IEA[5], is the chosen source of information, the estimation steps used to assess the total downstream EF have been presented below.

For the assessed exporting countries, only total downstream emission information is available. Downstream emissions consist of transmission and distribution emissions. For exporting countries, only transmission emissions are relevant for the total EF assessment. To separate the distribution emissions, Tier 1 EF from IPCC[17] were used and adjusted to estimate distribution emissions and thus evaluate transmission emission of the country. As a first approximation, Tier 1 EF range for gas distribution were adjusted using CL expertise to accurately reflect as best as possible the country's distribution infrastructure within the mathematical constraints of the equation (Distribution and transmission EF have to remain positive and in the range of the Tier 1 EF uncertainty when possible). The distribution emissions calculated in this step were removed from the total downstream emissions, to obtain the transmission emissions. Finally, the transmission emissions are divided by the volume of gas transmitted in billion cubic meters (bcm) to obtain the EF.

For countries with national data or data from academic paper, transmission emissions were directly available for the year of estimation. These emissions were divided by the total volume of gas transported, to obtain the downstream EF.

Calculating LNG related EF for exporting countries in CEF scenario. LNG related emissions include emissions from the LNG carrier (from country of production to the nearest port in Europe) and liquefaction. LNG carrier is the predominant source of emissions for LNG[18], and this has been estimated with a Carbon Limits' (CL) internal model. The CL LNG model is based on several scientific papers and developed in consultation with stakeholders in the maritime industry. The model used several assumptions for travel distance, time, idle days, etc. (Supplementary Tables 13 and 14). This model gives an emission factor for LNG Carrier. The estimates provided is by design quite generic and emissions from specific carriers may vary significantly.

Supplementary Table 13. Main assumptions considered in the LNG Carrier model

Field	*Value*
Number of days – at sea	Region specific – see Table 4
Number of days – idle	2.0
Number of days – loading	2.0
Boil off rate	0.15% per day

Supplementary Table 14. Number of days at sea for LNG carriers

Group of exporting countries	*Number of days*
North America: USA, Trinidad	8
South America: Peru	23
North Africa: Egypt, Algeria	4
West Africa: Nigeria, Angola	12
Australia	29
Russia	6
Norway	3
Qatar	17

There is very limited data on the liquefaction emissions. The average of 2019 emissions from all LNG liquefaction facilities where data was available was thus used as a proxy (Norway, the USA and Trinidad & Tobago – 7 facilities in total)[6-8]. For Norway, USA and Trinidad & Tobago, their country-specific facilities were used for emissions calculation.

Calculating downstream EF for importing countries in CEF scenario. For the importing region of Europe, only downstream (transmission and distribution) emissions and LNG unloading, and regasification related emissions are relevant for the total EF assessment (Supplementary Figure 14). For this region, an average EF for all European countries has been considered. The average downstream EF for all Europe (-8) has been calculated as the sum of transmission and distribution emissions using data from UNFCCC[9] and activity data from BP[19].

Calculating LNG related EF for importing countries in CEF scenario. For the importing region of Europe, assuming a proportional relationship between the LNG related emissions and LNG imported into the region, the regasification EF factor calculated by Marcogaz[10] has been used. Indeed, facing a lack of data about methane emission linked to LNG regasification this

industry paper which focused on the 21 large LNG import terminals in Europe has been used with a direct EF of 0.12 kt CH_4 per bcm of gas.

Calculating upstream and downstream EF in HP scenario. The harmonized pledges scenario considers country policies[20,21], Nationally Determined Contributions (NDC)[4] relevant for greenhouse gases (GHG) emission reduction and international pledges[22,23], to assess the changes in EF from the preliminary year (2019) to 2050. The International Pledges considered:

a. The "Zero Routine Flaring by 2030" initiative[22]: introduced by the World Bank, commits to (a) not routinely flare gas in new oil field developments and (b) to end routine flaring in existing (legacy) fields as soon as possible no later than 2030. For this initiative, this target has been assumed to be either reached by 2030 (optimistic) or by 2040 (delayed goal).

b. The "Methane Pledge" initiative[9]: participants joining the pledge agree to take voluntary actions to contribute to a collective effort to reduce global methane emissions at least 30% from 2020 levels by 2030. For this pledge, the 30% reduction in CH_4 emissions has been assumed to be country level target. This target is assumed to be either reached by 2040 (optimistic) or 2050 (delayed goal)

c. The "Methane Alliance" initiative[23], participants joining have two options as possible targets. In this case, either a methane intensity target of 0.25% is achieved by 2040 (optimistic) or a reduction target of 45% is achieved by 2050 (delayed goal).

    - Absolute reduction target of at least 45% reduction in methane emissions by 2025 and 60% to 75% by 2030.
    - Intensity target of "near-zero" methane emissions, targeting a CH_4 emission intensity of 0.25% or below.

Countries have been divided into four categories depending on their policies and participation in international pledges. Optimistic or delayed targets are set for each category of countries.

Category 1 countries: Countries with policy / NDC relevant for GHG emission reduction + international pledges. The following steps are applied

- Policy or NDC target is applied, by the target year
- Zero routine flaring by 2030 is applied alongside the pledges and/or NDC targets
- If both methane pledge and methane alliance pledges are signed, the optimistic target (Methane alliance) is implemented OR the target from the signed pledge is applied

Category 2 countries: Countries with policy / NDC relevant for GHG emission reduction & without any international pledges. The following steps are applied:

- Policy or NDC target is applied, by the target year

- EF is flat till 2050 from the year the target is achieved

Category 3 countries: Countries without policy / NDC relevant for GHG emission reduction & ONLY international pledges. The following steps are applied

- If 'Zero flaring by 2030' is signed, it is applied by 2040.
- If BOTH methane pledge and methane alliance are signed, the lowest target is applied by 2050 If one of the pledges is signed, the target is applied by 2050

Category 4 countries: Countries with current EF lower than BAT EF. EF remains flat from 2019 to 2050 in this case.

Calculating downstream and LNG related EF for importing countries in HP scenario. Country specific policies have not been directly considered for European countries but since GHG emissions reduction policies exist in European countries, adding to the fact that EF from the current scenario is already lower than the EF reported by European industries in the North of Europe – (including Norway), the EF remains flat from 2019 to 2050.

Calculating upstream and downstream EF in BAT scenario

For countries where IEA[5] is the main source of information used, for estimating upstream and downstream emissions in exporting countries, two abatement options are considered (Supplementary Figure 16).

Option 1: Use abatement values from IEA[24] and estimate emission reduction potential in 2030 and 2035, assuming that abatement options at no-net cost are achieved by 2030 and those with positive net-cost by 2035. IEA[24] provides the abatement potential by abatement type or technology, differentiating those with a negative net cost and those with a positive one. When the abatement potential can be applied for both oil and gas upstream part, the gas to oil ratio is used to estimate the potential abatement for gas; when the abatement potential is only relevant for oil, the APG to oil ratio is used to estimate APG related emission abatement potential. All the abatable emissions with negative net costs are subtracted from current emissions, for the year 2030, while the abatable emissions with positive abatement costs are subtracted from current emissions, for the year 2035. These emissions are divided by the activity data to obtain the EFs.

Option 2: Use OGCI industry target[25] for upstream CH_4 emissions for all countries. Converting the BAT industry target into EF - The OGCI provides upstream methane intensity targets: 0.25% by 2019, 0.20% by 2020, and well below 0.20% by 2025. In this option, this targeted EF achievable by the gas industries by 2019 and 2020 is assumed to be achieved by countries by 2030 and 2035 since EF calculated for 2019 were well above the 0.25% and 0.20% targeted EF. The industry targets of 0.25% and 0.20% have been converted from kt CH_4 emitted per kt CH_4 produced to kt CH_4 emitted per bcm of natural gas produced (90% of pure methane in the natural gas produced and a methane density of 667 kt per bcm has been assumed).

The higher of the two EFs is chosen as the upstream EF for 2030 and 2035. Following this, the target EF set by industries is applied by 2050. The EF between 2035 and 2050 is interpolated to find the EF in 2040 and 2045.

For countries where academic papers are available with country-based emission data, option 1 using abatement potential from IEA[5] and option 2 using BAT industry target are compared. In countries that have policies in place or under discussion for methane abatement, it is assumed the country can achieve the lowest EF target, hence the industry EF target is considered.

For countries where national data (such as UNFCCC[9]) is the chosen source of information, the calculated upstream and downstream EF in CEF was already below industry target. Hence, EF remains flat from 2019 until 2050. For the other countries, first, the European target of 29% reduction is applied by 2030, followed by applying OGCI target of 0.25% by 2030 and 0.20% by 2040. The EF remains flat from 2040 to 2050.

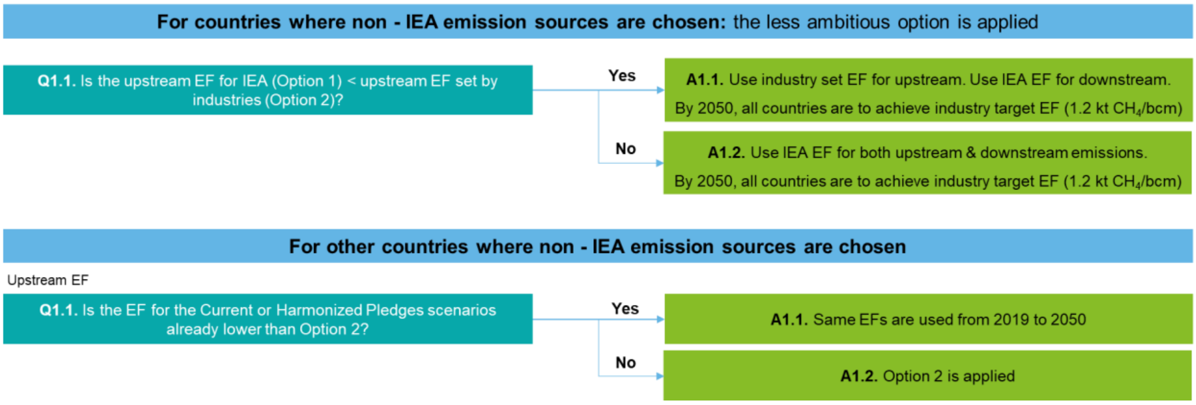

Supplementary Figure 16. Decision tree for selecting the BAT EF reduction trajectory. This figure is based on Hydrogen for Europe study design[1].

Calculating LNG EF in BAT scenario. Only the emissions associated with LNG carriers are updated in the BAT scenario due the lack of data for liquefaction and regasification facilities. Furthermore, a large portion of the LNG emissions come from the LNG carrier, making it one of the predominant segments for methane abatement. In this scenario, some changes have been made in the assumptions for 2050 used in the LNG carrier model developed by CL (Supplementary Table 15). EF from 2019 and 2050 are interpolated between the two values.

Supplementary Table 15. Main assumptions for the LNG Carrier model

Parameter	*Change*
Remaining BOG is	Reliquefied instead of sent to GCU
Engine load at sea loaded	90% instead of 80%
Engine load at sea ballast	60% instead of 40%
Boil off rate	already reduced according to new ship performances
Number of days idle	0 instead of 2
Share of BOG leaked	We trust them and assume 0% instead of 0.1%

Comparison of EF results between the three scenarios

Supplementary Figures 17 to 19 below show the calculated methane EFs for each of the BAT, CEF and HP scenarios.

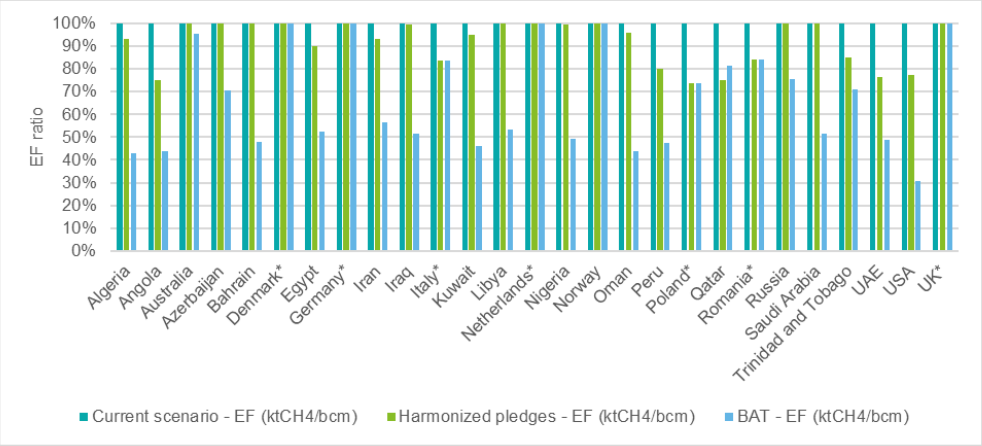

Supplementary Figure 17. EF (upstream, transmission, not including LNG EF) comparison between current emissions case, harmonized pledges, and BAT in 2030[1]. * EF also includes distribution.

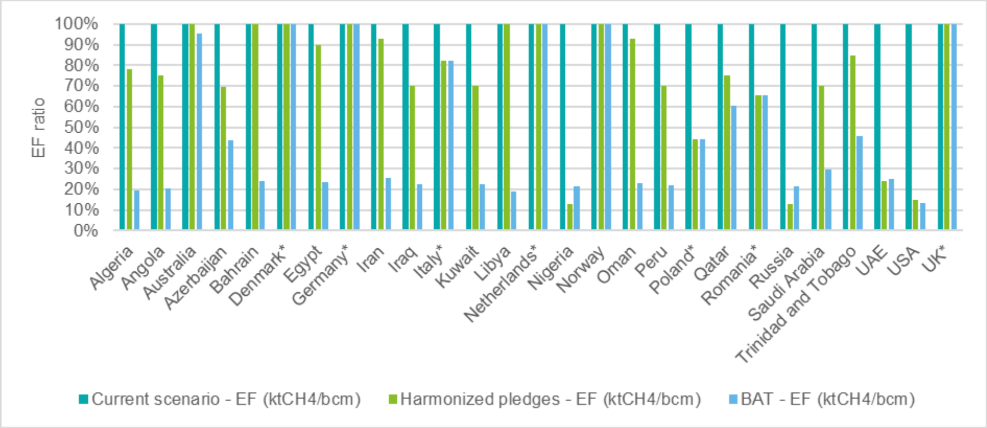

Supplementary Figure 18. EF (upstream, transmission, not including LNG EF) comparison between current emissions case, harmonized pledges, and BAT in 2040[1]. *EF also includes distribution.

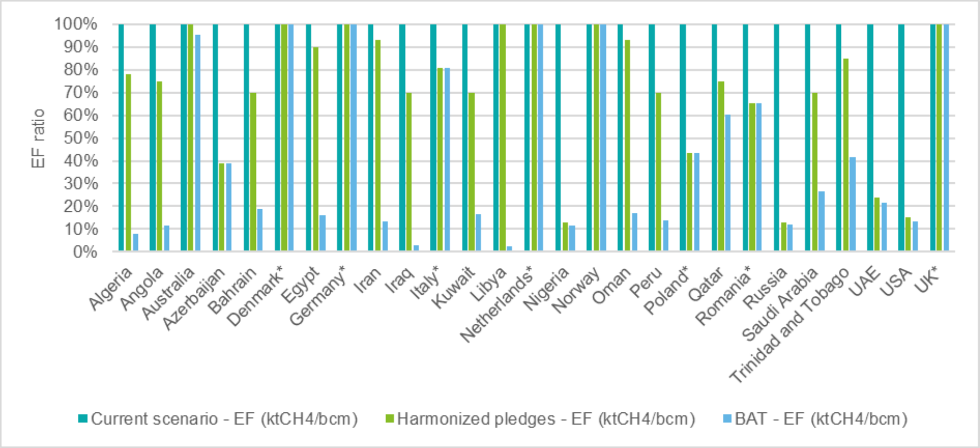

Supplementary Figure 19. EF (upstream, transmission, not including LNG EF) comparison between current emissions case, harmonized pledges, and BAT in 2050[1]. *EF also includes distribution.

Supplementary References

1. Hydrogen 4EU. *Charting Pathways to enable net zero* – 2022 edition (2022). https://www.hydrogen4eu.com/.

2. Alvarez, R.A., Zavala-Araiza, D., Lyon, D.R., Allen, D.T., Barkley, Z.R., Brandt, A.R., Davis, K.J., Herndon, S.C., Jacob, D.J., Karion, A., Kort, E.A., Lamb, B.K., Lauvaux, T., Maasakkers, J.D., Marchese, A.J., Omara, M., Pacala, S.W., Peischl, J., Robinson, A.L., Shepson, P.B., Sweeney, C., Townsend-Small, A., Wofsy, S.C., Hamburg, S.P.. Assessment of methane emissions from the U.S. oil and gas supply chain. *Science* 361, 186–188 (2018).

3. Norsk olje&gass. Methane emissions in Norway. Norsk olje&gass, Stavanger (2020). https://klimamiljorapport.norskoljeoggass.no/klima-og-milj%c3%b8rapport-2021/7-klimagassutslipp-og-andre-utslipp-til-luft/7-3-utslipp-av-klimagasser/7-3-6-utslipp-av-metan-ch4-

4. UNFCCC. *Nationally Determined Contributions Registry* (2022). https://unfccc.int/NDCREG.

5. IEA. *Global Methane Tracker* (2022). https://www.iea.org/reports/global-methane-tracker-2022

6. EPA. *Facility Level Information on GreenHouse gases Tool* (2022). https://ghgdata.epa.gov/ghgp/main.do.

7. Atlantic LNG. *Sustainability Report* (2018). https://atlanticlng.com/wp-content/uploads/2021/01/Atlantic-Sustainability-Report-2018.pdf

8. Equinor. *Onshore Facilities* (2021). https://www.equinor.com/energy/onshore-facilities.

9. European Commission & United States of America. *Global Methane Pledge* (2021). https://www.globalmethanepledge.org/#pledges.

10. UNFCCC. *GHG data from UNFCCC*. UNFCCC, Bonn, Germany (2021). https://unfccc.int/process-and-meetings/transparency-and-reporting/greenhouse-gas-data/ghg-data-unfccc/ghg-data-from-unfccc.

11. Norsk olje&gass. *Klima – og Miljorapport* (2021). https://klimamiljorapport.offshorenorge.no/klima-og-milj%C3%B8rapport-2021/7-klimagassutslipp-og-andre-utslipp-til-luft/7-3-utslipp-av-klimagasser/7-3-6-utslipp-av-metan-ch4-

12. Zhang, Y., Gautam, R., Pandey, S., Omara, M., Maasakkers, J., Sadavarte, P., Lyon, D., Nesser, H., Sulprizio, M., Varon, D., Zhang, R., Houweling, S., Zavala-Araiza, D., Alvarez, R., Lorente, A., Hamburg, S., Aben, I., Jacob, D. Quantifying methane emissions from the largest oil-producing basin in the United States from space. *Science Advances 6*, 5120. https://doi.org/10.1126/sciadv.aaz5120 (2020).

13. Ministero della transizione ecologica. *Produzione nazionale di idrocarburi* (2021). https://unmig.mise.gov.it/index.php/it/dati/ricerca-e-coltivazione-di-idrocarburi/produzione-nazionale-di-idrocarburi.

14. Danish Energy Agency. *Monthly and Yearly production* (2020). https://ens.dk/en/our-services/oil-and-gas-related-data/monthly-and-yearly-production.

15. Department for Business, Energy & Industrial Strategy. *Digest of UK Energy Statistics* (DUKES, 2020). https://www.gov.uk/government/statistics/digest-of-uk-energy-statistics-dukes-2021#:~:text=The%20Digest%20of%20United%20Kingdom,of%20energy%20as%20a%20whole.

16. IEA. *Putting gas flaring in the spotlight* (2020). https://www.iea.org/commentaries/putting-gas-flaring-in-the-spotlight.

17. IPCC. refinement to the 2006 IPCC Guidelines for the national GHGI, Volume 2 (2019). https://www.ipcc-nggip.iges.or.jp/public/2019rf/vol2.html.

18. Marcogaz. *Survey Methane Emissions for LNG Terminals in Europe* (2018). https://www.marcogaz.org/publications/survey-methane-emissions-for-lng-terminals-in-europe/.

19. BP. *Statistical Review of World Energy* (2021). https://www.bp.com/en/global/corporate/energy-economics/statistical-review-of-world-energy.html.

20. European Commission. Communication From The Commission To The European Parliament, The Council, *The European Economic And Social Committee And The Committee Of The Regions on an EU strategy to reduce methane emissions*, *European Commission*, Brussels, Belgium (2020). https://energy.ec.europa.eu/system/files/2020-10/eu_methane_strategy_0.pdf.

21. The White House Office of Domestic Climate Policy. *U.S. Methane Emissions Reduction Action Plan, The White House Office of Domestic Climate Policy*, Washington (2021). https://www.whitehouse.gov/wp-content/uploads/2021/11/US-Methane-Emissions-Reduction-Action-Plan-1.pdf.

22. The World Bank Group. *Zero Routine Flaring by 2030* (2015). The World Bank Group. https://www.worldbank.org/en/programs/zero-routine-flaring-by-2030.

23. Climate and Clean Air Coalition. *Global Methane Alliance*, *Climate and Clean Air Coalition*. Paris, France (2019). https://www.ccacoalition.org/en/activity/global-methane-alliance.

24. IEA. *Methane Tracker Data Explorer* (2022). https://www.iea.org/data-and-statistics/data-tools/methane-tracker-data-explorer.

25. OGCI. *Aiming for zero methane emissions initiative*. OGCI, London (2022). https://www.ogci.com/action-and-engagement/reducing-methane-emissions/.
